# Machine Learning Approaches to Predict 6-Month Mortality Among Patients With Cancer

**DOI:** 10.1001/jamanetworkopen.2019.15997

**Published:** 2019-10-25

**Authors:** Ravi B. Parikh, Christopher Manz, Corey Chivers, Susan Harkness Regli, Jennifer Braun, Michael E. Draugelis, Lynn M. Schuchter, Lawrence N. Shulman, Amol S. Navathe, Mitesh S. Patel, Nina R. O’Connor

**Affiliations:** 1 Department of Medicine, Perelman School of Medicine, University of Pennsylvania, Philadelphia; 2 Abramson Cancer Center, University of Pennsylvania, Philadelphia; 3 Penn Center for Cancer Care Innovation, University of Pennsylvania, Philadelphia; 4 Department of Medical Ethics and Health Policy, University of Pennsylvania, Philadelphia; 5 Corporal Michael J. Crescenz VA Medical Center, Philadelphia, Pennsylvania; 6 Penn Medicine, University of Pennsylvania, Philadelphia

## Abstract

**Importance:**

Machine learning algorithms could identify patients with cancer who are at risk of short-term mortality. However, it is unclear how different machine learning algorithms compare and whether they could prompt clinicians to have timely conversations about treatment and end-of-life preferences.

**Objectives:**

To develop, validate, and compare machine learning algorithms that use structured electronic health record data before a clinic visit to predict mortality among patients with cancer.

**Design, Setting, and Participants:**

Cohort study of 26 525 adult patients who had outpatient oncology or hematology/oncology encounters at a large academic cancer center and 10 affiliated community practices between February 1, 2016, and July 1, 2016. Patients were not required to receive cancer-directed treatment. Patients were observed for up to 500 days after the encounter. Data analysis took place between October 1, 2018, and September 1, 2019.

**Exposures:**

Logistic regression, gradient boosting, and random forest algorithms.

**Main Outcomes and Measures:**

Primary outcome was 180-day mortality from the index encounter; secondary outcome was 500-day mortality from the index encounter.

**Results:**

Among 26 525 patients in the analysis, 1065 (4.0%) died within 180 days of the index encounter. Among those who died, the mean age was 67.3 (95% CI, 66.5-68.0) years, and 500 (47.0%) were women. Among those who were alive at 180 days, the mean age was 61.3 (95% CI, 61.1-61.5) years, and 15 922 (62.5%) were women. The population was randomly partitioned into training (18 567 [70.0%]) and validation (7958 [30.0%]) cohorts at the patient level, and a randomly selected encounter was included in either the training or validation set. At a prespecified alert rate of 0.02, positive predictive values were higher for the random forest (51.3%) and gradient boosting (49.4%) algorithms compared with the logistic regression algorithm (44.7%). There was no significant difference in discrimination among the random forest (area under the receiver operating characteristic curve [AUC], 0.88; 95% CI, 0.86-0.89), gradient boosting (AUC, 0.87; 95% CI, 0.85-0.89), and logistic regression (AUC, 0.86; 95% CI, 0.84-0.88) models (*P *for comparison = .02). In the random forest model, observed 180-day mortality was 51.3% (95% CI, 43.6%-58.8%) in the high-risk group vs 3.4% (95% CI, 3.0%-3.8%) in the low-risk group; at 500 days, observed mortality was 64.4% (95% CI, 56.7%-71.4%) in the high-risk group and 7.6% (7.0%-8.2%) in the low-risk group. In a survey of 15 oncology clinicians with a 52.1% response rate, 100 of 171 patients (58.8%) who had been flagged as having high risk by the gradient boosting algorithm were deemed appropriate for a conversation about treatment and end-of-life preferences in the upcoming week.

**Conclusions and Relevance:**

In this cohort study, machine learning algorithms based on structured electronic health record data accurately identified patients with cancer at risk of short-term mortality. When the gradient boosting algorithm was applied in real time, clinicians believed that most patients who had been identified as having high risk were appropriate for a timely conversation about treatment and end-of-life preferences.

## Key Points

**Question:**

Can machine learning algorithms identify oncology patients at risk of short-term mortality to inform timely conversations between patients and physicians regrading serious illness?

**Findings:**

In this cohort study of 26 525 patients seen in oncology practices within a large academic health system, machine learning algorithms accurately identified patients at high risk of 6-month mortality with good discrimination and positive predictive value. When the gradient boosting algorithm was applied in real time, most patients who were classified as having high risk were deemed appropriate by oncology clinicians for a conversation regarding serious illness.

**Meaning:**

In this study, machine learning algorithms accurately identified patients with cancer who were at risk of 6-month mortality, suggesting that these models could facilitate more timely conversations between patients and physicians regarding goals and values.

## Teaser

This cohort study develops, validates, and compares machine learning algorithms that use structured electronic health record data before a clinic visit to predict mortality among patients with cancer.

## Introduction

Among patients with cancer, early advance care planning conversations lead to care that is concordant with patients’ goals and wishes, particularly at the end of life.^1,2^ Nevertheless, most patients with cancer die without a documented conversation about their treatment goals and end-of-life preferences and without the support of hospice care.^3,4,5,6^ A key reason for the dearth of such conversations may be that oncology clinicians cannot accurately identify patients at risk of short-term mortality using existing tools.^7,8^ Prognostic uncertainty and optimism bias may lead patients and clinicians to overestimate life expectancy, which can delay important conversations.^9,10,11,12,13^ While prognostic aids may inform better prognoses, existing prognostic aids do not apply to all cancers,^14,15^ do not identify most patients who will die within 6 to 12 months,^16^ and require time-consuming data input.^17^

Recent advances in computational capacity and machine learning (ML) allow more accurate prognoses by modeling linear and nonlinear interactions among many variables.^18,19,20^ Machine learning algorithms based on electronic health record (EHR) data have been shown to accurately identify patients at high risk of short-term mortality in general medicine settings,^21,22^ and oncology-specific ML algorithms can accurately predict short-term mortality among patients starting chemotherapy (eTable 1 in the Supplement).^19,20^ However, there are several concerns about ML-based prognostic tools that limit clinical applicability in oncology. First, to our knowledge, no study has assessed whether novel ML prognostic algorithms improve on traditional regression models in the oncology setting. Second, it is unclear whether oncologists believe that patients flagged by ML algorithms are appropriate for conversations about goals and values.

We hypothesized that ML algorithms could accurately identify all patients with cancer who are at risk of short-term mortality and that clinicians would believe that most patients who had been identified as high risk by the algorithm were appropriate for a conversation about goals and values. We developed, validated, and compared 3 ML models to estimate 6-month mortality among patients seen in oncology clinics affiliated with a large academic cancer center. We then assessed the feasibility of using real-time ML predictions in a community oncology practice to flag patients who may benefit from timely conversations about goals and values.

## Methods

### Data Source

We derived our cohort from patients receiving care at medical oncology clinics at the University of Pennsylvania Health System (UPHS) who were listed in Clarity, an Epic reporting database that contains individual EHRs for patients, including data on demographic characteristics, comorbidities, and laboratory results. Health insurance claim data were not available for this study. Our study followed the Transparent Reporting of a Multivariable Prediction Model for Individual Prognosis or Diagnosis (TRIPOD) reporting guideline for prediction model development and validation.^23^ This project was determined to qualify as quality improvement by the University of Pennsylvania institutional review board; need for informed consent was waived.

### Study Population

To develop our model, the cohort consisted of patients 18 years or older who had outpatient encounters with the listed specialties of oncology or hematology/oncology at 1 of 11 UPHS outpatient sites between February 1, 2016, and July 1, 2016. Patients were not required to have received cancer-directed treatment to be included in this study.

### Features

Our data set included 3 broad classes of variables (ie, features) that are commonly available in EHRs: (1) demographic variables (eg, age and sex); (2) Elixhauser comorbidities,^24^ and (3) laboratory and select electrocardiogram data. To transform raw EHR data into variables in our prediction model, we collected a complete history of every diagnosis code assigned to the patient prior to the encounter in question. Using *International Classification of Diseases, Ninth Revision *(*ICD*-*9*) and *ICD*-*10*, all diagnosis codes were categorized into 31 Elixhauser comorbidities (eMethods in the Supplement); 1 991 473 of 3 814 582 *ICD*-*9* and *ICD*-*10* diagnosis codes (52.2%) were classified as an Elixhauser comorbidity. For each encounter, we generated counts of the number of times each Elixhauser condition was ever coded (ie, total count) before the index encounter date. The purpose of generating a total count was to account for long-standing comorbidities before the index encounter that may have prognostic significance. To account for differential presence in the UPHS system and the development of more acute conditions, we generated counts of Elixhauser codes in the 180 days before the index encounter date (ie, recent count).

We analyzed all laboratory data in the 180 days before the index encounter date; only the 100 most common laboratory result types (listed by result name) were used in the models. For each laboratory result type, the following features were generated: proportion of results that were ordered as stat (range, 0-1), count of unique results, minimum and maximum values, SD of values, first recorded laboratory result, and last recorded laboratory result. No comorbidity or laboratory data after the index encounter date were included in model predictions.

All missing variables in the training set were imputed as 0 for count variables and using median imputation (ie, missing values were replaced by the median of all values) for noncount variables.^25^ The primary outcome of death was not included in the prediction model.

For all variables, we used several standard feature selection strategies, including dropping 0-variance features and highly correlated variables (eMethods in the Supplement). This process arrived at 559 features to include in all models (eTable 2 in the Supplement).

### Outcome

The primary outcome was 180-day mortality from the date of the encounter at an oncology practice. Date of death was derived from the first date of death recorded in either the EHR (from the Clarity database) or the Social Security Administration (SSA) Death Master File, matched to UPHS patients by social security number and date of birth. The SSA Death Master File contains information on the death of anyone holding a social security number as reported by relatives, funeral directors, financial institutions, and postal authorities.^26^ In a secondary analysis, we analyzed 500-day mortality from the encounter to determine the validity of the algorithms in identifying risk of long-term mortality.

### Machine Learning Algorithms

The study population was randomly split into a training cohort, in which the mortality risk algorithms were derived, and a validation cohort, in which the algorithms were applied and tested. The training cohort consisted of 70% of the UPHS cohort, and the validation cohort consisted of the remaining 30%. We randomly split our cohort at the patient level so that patients could not appear in both the training and validation sets. After random assignment, we selected 1 encounter per patient at random so that there was only 1 observation per patient in the training and validation sets. Patients were observed for up to 500 days after the index encounter. We derived 3 ML algorithms from the training data: a logistic regression model and 2 ensemble tree-based ML algorithms, ie, random forest and gradient boosted trees. We chose to use random forest and gradient boosting algorithms because they have been shown to identify patients at risk of short-term mortality based on structured EHR data and were easily trained using UPHS health records.^19,21^ We derived the logistic regression model using stepwise variable selection with backward elimination, resulting in a final model with 34 variables (eMethods in the Supplement). For the random forest and gradient boosting algorithms, hyperparameters were determined by using a grid search and 5-fold cross-validation on the training cohort to determine the values that led to the best performance. Further details on the ML models are presented in the eMethods in the Supplement. We did not recalibrate models after validation. All data and code are publicly available.^27^

### Variable Importance

Variable importance was determined by the coefficient absolute value for the logistic regression model and selection frequency for the random forest and gradient boosting models. The technique of using selection frequency to determine variable importance in ML models has been described previously.^22^

### Clinical Feasibility Assessment

To determine the feasibility of an ML model prompting conversations about goals and values, we created weekly lists of patients with 30% or greater risk of 6-month mortality based on predictions from the gradient boosting algorithm for 1 UPHS community-based general hematology/oncology practice. We chose to present the gradient boosting model a priori because previous analyses have suggested that such models have high area under the receiver operating characteristic curve (AUC) and positive predictive value (PPV) for predicting 6-month mortality.^19^ To generate real-time predictions, we used an older version of the gradient boosting model that did not incorporate robust feature selection or hyperparameter optimization but was part of our initial clinical feasibility testing; performance characteristics of this older model are reported in eTable 3 in the Supplement. We chose the 30% risk threshold based on expert consensus from the clinicians in the study and a previous analysis of a similar algorithm used to help direct inpatient palliative care consults.^28^ For 4 consecutive weeks in October 2018, we provided 15 clinicians with printed lists of high-risk oncology patients in the practice who had been identified as having high risk by the algorithm and had appointments in the upcoming week. At a weekly practice meeting, clinicians indicated yes or no for each patient appointment in the upcoming week to indicate whether that patient was appropriate for a conversation about goals and end-of-life preferences. For clinicians who completed the survey, we calculated proportions of patients identified as having high risk who were indicated as appropriate for such conversations. The Wilcoxon rank sum test was used to compare predicted 6-month mortality risk between patients deemed as appropriate vs others on the high-risk lists.

### Statistical Analysis

We used descriptive statistics to compare the characteristics of the study population, stratified by death status (ie, alive or deceased) at 6 months. Algorithms were developed from the training cohort and assessed on the independent validation cohort, which played no role in model development, by calculating the PPV and AUC. As the PPV varies by risk threshold, we set the alert rate (ie, the proportion of patient encounters flagged in the validation set) to 0.02 for each model and derived the PPV and all other threshold-dependent performance metrics at this alert rate. Because PPV is threshold dependent, we also compared models using the AUC, ie, the probability that a randomly selected patient who dies during the follow-up period will have a higher risk score than a patient who did not die. We chose to present the AUC because it is a threshold-independent measure of discrimination. A 95% CI for each AUC was estimated using bootstrapping.^29^ To compare AUCs among models, we used a permutation test with pairwise comparisons,^30^ using the Bonferroni method to adjust for multiple comparisons. Statistical significance for primary analysis was set at *P* < .05; following Bonferroni correction, it was set at *P* < .017. All tests were 2-tailed.

To further describe model performance, we also constructed model calibration plots and calculated secondary metrics of clinical prediction models, including accuracy and specificity. All analyses were conducted using the Sklearn version 0.15.2 package^31^ in Python (Python Software Foundation) and occurred between October 1, 2018, and September 1, 2019.

## Results

### Cohort Characteristics

There were a total of 62 377 encounters during the study period among 26 525 patients, which represented the analytic cohort. The training and validation cohorts consisted of 18 567 (70.0%) and 7958 (30.0%) unique patients, respectively (eFigure 1 in the Supplement).

### Study Population Characteristics

Of 26 525 patients in the training and validation cohorts, 1065 (4.0%) died during the 180-day follow-up period. Patients alive at 6 months were significantly more likely to be female (15 922 [62.5%] vs 500 [47.0%]; *P* < .001) and younger (mean age, 61.3 [95% CI, 61.1-61.5] years vs 67.3 [95% CI, 66.5-68.0] years; *P* < .001) than patients who died at 6 months, although there was no significant different in race. All characteristics, including selected comorbidities and laboratory values, are presented in Table 1. Full distributions of comorbidities and laboratory values are presented in eTable 4 and eTable 5 in the Supplement.

**Table 1.** Patient Characteristics, Stratified by Death Status Within 6 Months of the Index Encounter

Characteristic	No. (%)	
	Alive at 6 mo (n = 25 460)	Died at 6 mo (n = 1065)
Age, mean (95% CI), y	61.3 (61.1-61.5)	67.3 (66.5-68.0)
Race/ethnicity		
White	18 920 (74.3)	767 (72.0)
Black	4163 (16.4)	191 (17.9)
Asian	535 (2.1)	16 (1.5)
Hispanic, white	346 (1.4)	14 (1.3)
Hispanic, black	96 (0.4)	3 (0.3)
East Indian	83 (0.3)	1 (0.1)
Pacific Islander	38 (0.1)	2 (0.2)
American Indian	28 (0.1)	2 (0.2)
Other	584 (2.3)	30 (2.8)
Unknown	659 (2.6)	39 (3.7)
Women	15 922 (62.5)	500 (47.0)
Selected comorbidities		
Hypertension	8600 (33.8)	472 (44.3)
Renal failure	1891 (7.4)	151 (14.2)
COPD	3631 (14.3)	227 (21.3)
Congestive heart failure	1536 (6.0)	141 (13.2)
Fluid and electrolyte disorders	4526 (17.8)	417 (39.2)
Most recent laboratory values, mean (95% CI)		
Hemoglobin, g/dL	12.2 (12.1-12.2)	11.0 (10.9-11.1)
Platelets, ×10^3^/μL	227.1 (226.1-228.1)	229.8 (222.4-237.3)
White blood cells, /μL	7.0 (6.9-7.1)	8.0 (7.6-8.4)
Creatinine, mg/dL	0.95 (0.93-0.98)	1.03 (0.98-1.08)
Total calcium, mg/dL	9.3 (9.3-9.3)	9.2 (9.1-9.2)
ALT, U/L	20.0 (19.7-20.2)	26.7 (24.3-29.0)
Total bilirubin, mg/dL	0.55 (0.55-0.56)	0.83 (0.70-0.97)
Alkaline phosphatase, U/L	77.1 (76.6-77.7)	122.3 (114.5-130.0)
Albumin, g/dL	4.0 (4.0-4.0)	3.7 (3.6-3.7)

Abbreviations: ALT, alanine aminotransferase; COPD, chronic obstructive pulmonary disease.

SI conversion factors: To convert hemoglobin to g/L, multiply by 10.0; platelet count to ×10^9^/L, multiply by 1.0; white blood cell count to ×10^9^/L, multiply by 0.001; creatinine to μmol/L, multiply by 76.25; total calcium to mmol/L, multiply by 0.25; ALT to μkat/L, multiply by 0.0167; total bilirubin to μmol/L, multiply by 17.104; alkaline phosphatase to μkat/L, multiply by 0.0167; and albumin to g/L, multiply by 10.

### Algorithm Variable Importance

The top 10 variables in terms of variable importance for the 3 algorithms are shown in the Box. The top predictors shared across all models were most recent albumin and alkaline phosphatase levels and number of recent and total diagnostic codes for solid tumor without metastasis and metastatic cancer. Sex, total or direct bilirubin, creatinine, and hemoglobin did not have high importance in any model. A broader listing of variable importance can be found in eTable 6 in the Supplement.

Box. **Variable Importance in Descending Order of Coefficient Effect Size for Logistic Regression Model or Feature Importance for Random Forest and Gradient Boosting Models^a^**

**Logistic Regression**

Albumin, last laboratory value
Solid tumor, recent count of diagnostic codes
Metastatic cancer, recent count of diagnostic codes
Patient age
Alkaline phosphatase, last laboratory value
Gender
Solid tumor, total count of diagnostic codes
Blood loss anemia, total count of diagnostic codes
Red blood cells, last laboratory value
MCHC, last laboratory value

**Random Forest**

Metastatic cancer, recent count of diagnostic codes
Albumin, last laboratory value
Alkaline phosphatase, last laboratory value
Albumin, minimum laboratory value
Patient age
Alkaline phosphatase, maximum laboratory value
Solid tumor, total count of diagnostic codes
Solid tumor, recent count of diagnostic codes
Metastatic cancer, total count of diagnostic codes
Lymphocytes, %, minimum laboratory value

**Gradient Boosting**

Albumin, last laboratory value
Solid tumor, recent count of diagnostic codes
Metastatic cancer, total count of diagnostic codes
Metastatic cancer, recent count of diagnostic codes
Alkaline phosphatase, last laboratory value
Lymphocytes, %, last laboratory value
Neutrophils, %, last laboratory value
Albumin, minimum laboratory value
Alkaline phosphatase, maximum laboratory value
Lymphocytes, %, minimum laboratory value

Abbreviation: MCHC, mean corpuscular hemoglobin concentration.

^a^Variable importance is ranked by absolute value of coefficient for logistic regression model and by selection frequency for the random forest and gradient boosting models.

### Model Performance

Algorithm discrimination and other performance metrics in the validation set are presented for each model in Table 2. At the prespecified alert rate, the random forest and gradient boosting models had higher PPVs (51.3% and 49.4%, respectively) than the logistic regression model (44.7%). After adjusting for multiple comparisons, there was no significant difference in AUC among the random forest (0.88; 95% CI, 0.86-0.89), gradient boosting (0.87; 95% CI, 0.85-0.89), and logistic regression (0.86; 95% CI, 0.84-0.88) models (*P *for comparison = .02). All models had accuracy of 95% or higher and specificity of 98.9% or higher. Despite hyperparameter tuning, the random forest and gradient boosting algorithms both displayed evidence of overfitting, with AUCs in the training set of 0.98 and 0.94, respectively.

**Table 2.** Performance Metrics of Machine Learning Models^a^

Algorithm	Positive Predictive Value^b^	AUC^b^	Accuracy	Specificity
Random forest	0.513^c^	0.88^c^	0.96^c^	0.99^c^
Gradient boosting classifier	0.494	0.87	0.96^c^	0.99^c^
Logistic regression	0.447	0.86	0.95	0.99^c^

Abbreviation: AUC, area under the receiver operating characteristic curve.

^a^ Positive predictive value, accuracy, and specificity were determined by setting the alert rate in the test set for each algorithm to 0.02. At this prespecified alert rate, the 6-month mortality risk threshold was 0.27 for the random forest model; 0.15 for the gradient boosting model; and 0.33 for the logistic regression model.

^b^ Coprimary performance metric.

^c^ Refers to the best-performing model(s) for each performance metric.

Model calibration plots for the 3 models appear in eFigure 2 in the Supplement. The logistic regression and random forest models were well calibrated for patients with low probabilities of death. When the probability of death was greater than 30%, logistic regression generally overestimated the risk of death and random forest significantly underestimated risk of death. The gradient boosting model systematically underestimated risk of death.

The observed survival in the 180 days after the initial encounter appears in the Figure, stratified by patients at high risk vs patients at low risk, as identified by the random forest model. In sensitivity analyses, patients at high risk had a much lower observed 180-day survival across varying thresholds of predicted risk (eFigure 3 in the Supplement). At a prespecified alert rate of 0.02 in the random forest model (corresponding to a 180-day mortality risk of 27%), observed 180-day mortality was 51.3% (95% CI, 43.6%-58.8%) in the group at high risk vs 3.4% (95% CI, 3.0%-3.8%) in the group at low risk. These differences persisted even when observing patients 500 days after the index encounter: observed 500-day mortality was 64.4% (95% CI, 56.7%-71.4%) in the group at high risk vs 7.6% (95% CI, 7.0%-8.2%) in the group at low risk (eFigure 4 in the Supplement).

**Figure.**
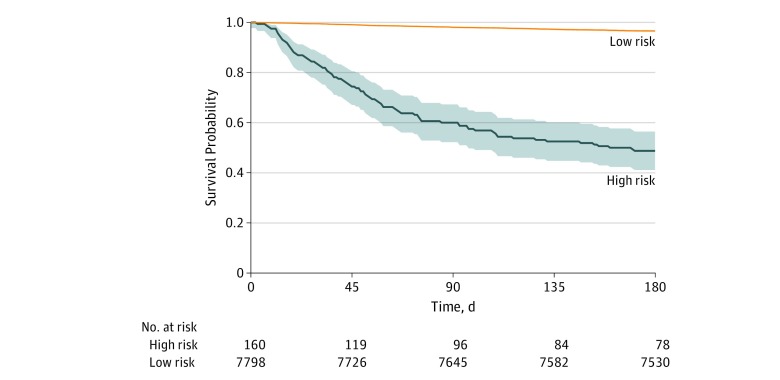
Observed 180-Day Survival for Random Forest Model Risk threshold was determined in the random forest model by setting the alert rate to 0.02, which corresponds to a proportion risk of 180-day mortality of 27%. Shaded areas indicate 95% CIs.

### Feasibility of Informing Conversations

In a survey of 15 providers at a community-based hematology/oncology practice, clinicians assessed 171 of the 328 potential high-risk encounters identified by an older version of the gradient boosting algorithm. Of the 15 clinicians, 2 completed the survey all 4 weeks assessed; 9 completed the survey 2 to 3 of the 4 survey dates; and 4 completed the survey once during the 4 weeks (survey response rate, 52.1%). Of 171 patients assessed, 100 unique patients (58.8%) were indicated as appropriate for a conversation about goals and preferences in the upcoming week. The mean predicted 6-month mortality risk of all high-risk encounters was 0.65. There was no difference in mortality risk between patients identified as appropriate vs others on the high-risk list (mean 6-month mortality risk, 0.67 vs 0.64; *P* = .15).

## Discussion

In this cohort study, ML models based on structured EHR data accurately predicted the short-term mortality risk of individuals with cancer from oncology practices affiliated with an academic cancer center. The gradient boosting and random forest models had good PPV at manageable alert rates, and all ML models had adequate discrimination (ie, AUC, 0.86-0.88) in predicting 6-month mortality. The PPVs of the random forest and gradient boosting algorithms were much higher than historical estimates from clinician assessment alone.^7,16^ Unlike standard prognostic tools, our models incorporated variability in laboratory data and many comorbidities into predictions. Moreover, clinicians expressed reasonable agreement that the patients determined to have the highest predicted risk of death by 1 of the ML models were appropriate for a conversation about goals and end-of-life preferences, an early indication that ML-derived mortality predictions may be useful for encouraging these discussions.

There are several strengths of this analysis. To our knowledge, this is the first investigation comparing ML classifiers, including regression-based classifiers, to predict mortality in a large general oncology population.^32^ Unlike previously developed ML-based prognostic tools in oncology,^19,20^ our models were trained on all patients seen at oncology or hematology/oncology practices regardless of receipt of cancer-directed therapy. Because some patients could have received care outside of the UPHS system and we did not have access to registry or claims data, we could not assess what proportion of our cohort received systemic therapy after the index encounter. Furthermore, compared with previously published ML classifiers in oncology, our models used fewer variables, all of which are commonly available in structured formats in real-time EHR databases. Thus, this model is more efficient than previously trained ML models in the general oncology setting. Finally, most patients identified as having high risk by the model were deemed appropriate for a conversation about goals and end-of-life preferences by oncology clinicians. Our survey findings should be interpreted with some caution because we used an older version of the gradient boosting model with less robust feature selection and hyperparameter optimization. Using the fully optimized version of the gradient boosting or random forest models, which had a higher PPV than the version presented to clinicians during the survey, may have improved results from the survey.

Machine learning classifiers, in contrast to regression-based classifiers, account for often unexpected predictor variables and interactions and can facilitate recognition of predictors not previously described in the literature.^32,33^ All models had excellent discriminative performance and PPV for predicting 6-month mortality, particularly compared with other EHR-based gradient boosting and random forest machine prognostic models published in the literature.^19,21^

In contrast to previous reports,^21^ there was no statistically significant difference in AUC among the gradient boosting, random forest, and logistic regression algorithms after adjusting for multiple comparisons, although the random forest model had an advantage compared with the logistic regression model. However, the gradient boosting and random forest models outperformed the logistic regression model in PPV, which is potentially more clinically relevant than AUC.^34^ Finally, all models placed importance on variables with known prognostic implications, including age, diagnosis of metastatic cancer, most recent albumin level, and most recent alkaline phosphatase level. The regression model tended to place more importance on diagnosis codes and demographic characteristics than the random forest or gradient boosting models, which placed more importance on recent laboratory values.

Accurate identification of patients at high risk of short-term mortality is important in oncology given the release of recent guidelines advocating for early palliative care and advance care planning for high-risk populations.^3,4^ Our findings demonstrated that ML algorithms can predict a patient’s risk of short-term mortality with good discrimination and PPV. Such a tool could be very useful in aiding clinicians’ risk assessments for patients with cancer as well as serving as a point-of-care prompt to consider discussions about goals and end-of-life preferences. Machine learning algorithms can be relatively easily retrained to account for emerging cancer survival patterns. As computational capacity and the availability of structured genetic and molecular information increase, we expect that predictive performance will increase and there may be a further impetus to implement similar tools in practice.

### Limitations

There are several limitations to this analysis. First, even with robust feature selection and hyperparameter optimization, the random forest and gradient boosting models were overfit or fit peculiarities in the training data that may not generalize to other data sources. Despite overfitting, the gradient boosting and random forest models had excellent discrimination and good PPVs in the holdout validation set, outperforming the logistic regression model. Nevertheless, these models should be validated in other oncology settings to determine generalizability.

Second, unlike previous analyses comparing ML approaches with routinely used predictive tools,^22,33^ there was not a criterion-standard prognostic assessment tool for comparison, and it is unclear whether our models outperformed other previously described tools in disease-specific settings. A previous analysis found that ML predictions in specific subgroups outperformed predictions from randomized clinical trials or registry data.^19^ Our study was underpowered for these subgroup comparisons.

Third, these tools were developed to be used in a general medical oncology setting and may not be generalizable to patients seen in radiation oncology, gynecologic oncology, or other oncology specialty practices or health systems with different EHRs. However, the features used in our models are all commonly available in structured data fields in most health system EHRs.

Fourth, our primary outcome relied in part on SSA data, which are commonly used to determine mortality in health services research. It has recently been shown that the SSA Death Master File may underestimate actual mortality rates.^35^ We attempted to address this by supplementing SSA data with EHR death information; however, some misclassification may still exist.

Fifth, our survey only assessed the feasibility of an ML model prompting serious illness conversations and was not a definitive validation of model performance. Clinicians may have had practical reasons for indicating that high-risk patients were not appropriate for serious illness conversations, including known patient and family preferences that would have precluded a conversation that week. Furthermore, we only surveyed clinicians regarding patients identified as having high risk and thus could have inadvertently biased clinicians toward responding that patients were appropriate for a conversation about goals and end-of-life wishes.

## Conclusions

This cohort study demonstrated that, in a large heterogeneous population of patients seeking outpatient oncology care, ML algorithms based on structured real-time EHR data had adequate performance in identifying outpatients with cancer who had high risk of short-term mortality. According to clinician surveys, most patients flagged as having high risk by one of the ML models were appropriate for a timely conversation about goals and end-of-life preferences. Our findings suggest that ML tools hold promise for integration into clinical workflows to ensure that patients with cancer have timely conversations about their goals and values.

## Notes

Supplement. **eMethods.** Codes, Regression Output, and Model Parameters **eReferences.** **eFigure 1. **Cohort Selection Process **eFigure 2. **Model Calibration Plots **eFigure 3. **Observed 180-Day Survival by Varying Risk Thresholds for Random Forest Model **eFigure 4. **Observed 500-Day Survival by Predicted Risk for Random Forest Model **eTable 1. **Published Electronic Health Record-Based Machine Learning Prognostic Tools **eTable 2. **Variables Included in Machine Learning Algorithms **eTable 3. **Performance Characteristics of Gradient Boosting Model Used for Clinician Surveys **eTable 4. **Distribution of Coded Elixhauser Comorbidities **eTable 5. **Baseline Laboratory and Electrocardiogram Values **eTable 6. **Variable Importance by Model, Top 20 Predictors
