# Supplementary material for: Machine Learning Approaches to Predict 6-Month Mortality Among Patients With Cancer
Source: JAMA Netw Open. 2019 Oct 25;2(10):e1915997. doi: 10.1001/jamanetworkopen.2019.15997 (PMC6822091; doi:10.1001/jamanetworkopen.2019.15997)
Supplement: Supplement. — eMethods. Codes, Regression Output, and Model Parameters eReferences. eFigure 1. Cohort Selection Process eFigure 2. Model Calibration Plots eFigure 3. Observed 180-Day Survival by Varying Risk Thresholds for Random Forest Model eFigure 4. Observed 500-Day Survival by Predicted Risk for Random Forest Model eTable 1. Published Electronic Health Record-Based Machine Learning Prognostic Tools eTable 2. Variables Included in Machine Learning Algorithms eTable 3. Performance Characteristics of Gradient Boosting Model Used for Clinician Surveys eTable 4. Distribution of Coded Elixhauser Comorbidities eTable 5. Baseline Laboratory and Electrocardiogram Values eTable 6. Variable Importance by Model, Top 20 Predictors [file jamanetwopen-2-e1915997-s001.pdf]

Supplementary Online Content

Parikh RB, Manz C, Chivers C, et al. Machine learning approaches to predict 6-month mortality among patients with cancer. *JAMA Netw Open*. 2019;2(10):e1915997. doi:10.1001/jamanetworkopen.2019.15997

eMethods. Codes, Regression Output, and Model Parameters

eReferences.

eFigure 1. Cohort Selection Process

eFigure 2. Model Calibration Plots

eFigure 3. Observed 180-Day Survival by Varying Risk Thresholds for Random Forest Model

eFigure 4. Observed 500-Day Survival by Predicted Risk for Random Forest Model

eTable 1. Published Electronic Health Record-Based Machine Learning Prognostic Tools

eTable 2. Variables Included in Machine Learning Algorithms

eTable 3. Performance Characteristics of Gradient Boosting Model Used for Clinician Surveys

eTable 4. Distribution of Coded Elixhauser Comorbidities

eTable 5. Baseline Laboratory and Electrocardiogram Values

eTable 6. Variable Importance by Model, Top 20 Predictors

This supplementary material has been provided by the authors to give readers additional information about their work.

© 2019 Parikh RB et al. *JAMA Network Open.*

eMethods. Codes, Regression Output, and Model Parameters

I. Regular expression patterns for Elixhauser Comorbidities
II. Stepwise logistic regression output
III. Feature selection for all models
IV. Hyperparameter optimization for gradient boosting and random forest algorithm

© 2019 Parikh RB et al. *JAMA Network Open.*

I. Regular expression patterns for Elixhauser Comorbidities

<table>
<tr><th>Comorbidity</th><th>ICD-10 Regular Expression Pattern</th><th>ICD-9 Regular Expression Pattern</th><th>ICD-10 Codes Observed</th><th>ICD-9 Codes Observed</th></tr>
<tr><td>Alcohol abuse</td><td>['F10', 'E52', 'G621', 'I426', 'K292', 'K70[039]', 'T51', 'Z502', 'Z714', 'Z721']</td><td>['2652', '291[12356789]', '303[09]', '3050', '3575', '4255', '5353', '571[0123]', '980', 'V113']</td><td>['F10.10', 'T51.91XD', 'K70.30', 'K70.31', 'F10.21', 'F10.20', 'F10.239', 'T51.0X1A', 'F10.982', 'F10.231', 'K29.21', 'F10.129', 'F10.11', 'F10.99', 'K70.9', 'I42.6', 'F10.19', 'F10.96', 'F10.29', 'F10.288', 'K70.0']</td><td>['303.90', '305.00', '305.03', '291.81', '303.91', '303.93', '305.01', '291.2', '571.2', '571.3', 'V11.3', '303.00', '305.02', '425.5', '535.30', '571.1', '357.5', '303.01', '571.0', '980.0', '303.02', '291.89']</td></tr>
<tr><td>Cardiac arrhythmias</td><td>['I44[123]', 'I456', 'I459', 'I4[789]', 'R00[018]', 'T821', 'Z[49]50']</td><td>['426([079]|1[023])', '427[01234]', 'V450', 'V533']</td><td>['I47.1', 'I48.0', 'I48.91', 'I48.2', 'I44.2', 'I48.1', 'I49.8', 'I44.1', 'I49.5', 'I49.3', 'I48.92', 'R00.0', 'I49.1', 'R00.1', 'Z95.0', 'I47.2', 'I45.9', 'I45.6', 'I47.9', 'I48.3', 'I49.49', 'I48.4', 'T82.199A', 'T82.190A', 'I49.9', 'I49.01', 'Z45.010', 'I44.30', 'Z45.02', 'R00.8', 'I47.0', 'T82.198A', 'Z45.09', 'Z45.018', 'T82.119A', 'T82.119D', 'T82.110A', 'I44.39']</td><td>['427.0', '427.31', '427.1', '426.0', '426.13', 'V45.01', 'V45.02', 'V53.31', '426.12', '427.32', '426.9', '427.2', '427.41', '426.7', 'V53.32', '427.42', '426.10', 'V45.09', 'V53.39', 'V45.00']</td></tr>
<tr><td>Blood loss anemia</td><td>['D500']</td><td>['2800']</td><td>['D50.0']</td><td>['280.0']</td></tr>
<tr><td>Congestive heart failure</td><td>['I099', 'I1(10|3[02])', 'I255', 'I4(2[056789]|3)', 'I50', 'P290']</td><td>['39891', '402(01|11|91)', '404(01|03|[19][13])', '42(5[456789]|8)']</td><td>['I50.32', 'I50.33', 'I42.8', 'I42.9', 'I25.5', 'I50.23', 'I50.43', 'I50.30', 'I50.9', 'I50.21', 'I50.22', 'I50.42', 'I50.1', 'I50.31', 'I50.20', 'I50.40', 'I42.0', 'I13.2', 'I42.5', 'I50.41', 'I42.7', 'I43', 'I13.0', 'I50.810', 'I11.0', 'I09.9', 'I42.6']</td><td>['428.32', '428.0', '428.33', '425.4', '404.91', '428.43', '402.91', '428.30', '428.21', '425.9', '428.22', '425.8', '428.23', '402.11', '404.11', '428.1', '428.31', '428.42', '428.40', '425.7', '428.9', '398.91', '428.20', '428.41', '425.5', '404.01', '404.93', '404.03']</td></tr>
<tr><td>Chronic pulmonary disease</td><td>['I27[89]', 'J4[01234567]', 'J6([01234567]|84)', 'J70[13]']</td><td>['416[89]', '49', '50([012345]|64|8[18])']</td><td>['J44.9', 'J45.20', 'J43.8', 'J45.909', 'J45.30', 'J40', 'J45.40', 'J45.990', 'J47.1', 'J47.9', 'J67.9', 'J43.9', 'J41.0', 'J43.2', 'J45.50', 'J44.1', 'J45.21', 'J43.1', 'J45.901', 'J41.1', 'J42', 'J45.991', 'J44.0', 'J45.31', 'I27.9', 'J45.41', 'J45.51', 'I27.82', 'J70.1', 'I27.81', 'J43.0', 'J45.998', 'J41.8', 'J47.0', 'I27.89', 'J61', 'J45.22', 'J45.42', 'J63.2', 'J70.3', 'J45.32']</td><td>['416.9', '492.8', '496', '491.8', '416.8', '490', '493.90', '493.10', '493.92', '493.20', '493.22', '494.0', '508.1', '501', '493.81', '493.00', '492.0', '494.1', '491.0', '491.21', '491.20', '491.1', '491.9', '493.82', '493.02', '495.9', '493', '493.01', '493.9', '493.91', '493.21', '491.22', '500', '493.12', '495.8', '508.8', '505', '502', '503', '493.11', '495.2']</td></tr>
<tr><td>Coagulopathy</td><td>['D6[5678]', 'D69[13456]']</td><td>['286', '2871', '287[345]']</td><td>['D69.3', 'D69.6', 'D68.0', 'D68.8', 'D69.59', 'D68.4', 'D68.1', 'D68.61', 'D68.59', 'D68.51', 'D68.62', 'D67', 'D69.49', 'D68.9', 'D69.1', 'D68.69', 'D65', 'D68.2', 'D68.52', 'D69.41', 'D66', 'D68.311', 'D68.32']</td><td>['287.5', '287.31', '286.9', '286.4', '287.30', '287.49', '287.4', '286.2', '287.1', '287.3', '286.3', '286.1', '286.7', '286.6', '286.5', '287.32', '287.39', '286.0', '287.33', '286.59', '287.41', '286.53', '286.52']</td></tr>
<tr><td>Deficiency anemia</td><td>['D50[89]', 'D5[123]']</td><td>['280[123456789]', '281']</td><td>['D50.9', 'D50.8', 'D51.9', 'D51.0', 'D51.3', 'D51.8', 'D53.1', 'D53.9', 'D53.8', 'D53.2', 'D51.1', 'D53.0', 'D52.8', 'D52.9']</td><td>['280.9', '281.9', '281.1', '281.0', '280.8', '280.1', '281.3', '281.2']</td></tr>
</table>

© 2019 Parikh RB et al. *JAMA Network Open.*

Depression	['F204', 'F31[345]', 'F3[23]', 'F341', 'F4[13]2']	['296[235]', '3004', '309', '311']	['F32.9', 'F33.1', 'F33.41', 'F33.2', 'F43.23', 'F32.4', 'F32.1', 'F33.0', 'F34.1', 'F43.20', 'F32.3', 'F32.0', 'F32.5', 'F33.3', 'F31.30', 'F43.22', 'F43.21', 'F32.8', 'F32.2', 'F33.42', 'F33.40', 'F31.4', 'F43.29', 'F33.9', 'F31.32', 'F31.5', 'F43.24']	['311', '309.28', '300.4', '296.20', '296.32', '309.9', '309.0', '309.81', '296.30', '296.25', '296.31', '296.35', '296.36', '296.33', '296.22', '296.24', '296.23', '309.24', '309.83', '296.34', '296.55', '309.29', '296.21', '296.26', '309.1', '296.53', '296.50', '296.51', '296.52', '309.89', '296.56', '309.4', '296.54']
Diabetes complicated	['E1[01234][2345678]']	['250[456789]']	['E11.65', 'E11.329', 'E11.29', 'E11.22', 'E11.319', 'E11.8', 'E11.40', 'E11.69', 'E11.649', 'E11.21', 'E11.49', 'E10.29', 'E10.8', 'E11.359', 'E13.21', 'E11.621', 'E10.65', 'E11.3299', 'E10.649', 'E11.42', 'E13.40', 'E11.59', 'E11.39', 'E11.3599', 'E10.359', 'E11.311', 'E11.610', 'E11.622', 'E13.42', 'E10.319', 'E10.22', 'E11.43', 'E11.339', 'E11.618', 'E10.329', 'E10.3299', 'E13.65', 'E13.8', 'E11.351', 'E13.339', 'E11.321', 'E11.349', 'E11.3499', 'E11.628', 'E10.21', 'E10.42', 'E10.618', 'E11.52', 'E13.29', 'E10.43', 'E10.40', 'E11.41', 'E13.359', 'E10.36', 'E11.51', 'E11.341', 'E11.3419', 'E11.331', 'E11.3319', 'E13.3599', 'E13.311', 'E11.36', 'E13.51', 'E13.319', 'E10.49', 'E13.329', 'E13.628', 'E13.43', 'E13.621', 'E10.59', 'E11.44']	['250.40', '250.50', '250.60', '250.80', '250.41', '250.51', '250.42', '250.62', '250.82', '250.81', '250.92', '250.90', '250.52', '250.61', '250.83', '250.70', '250.9', '250.93', '250.71', '250.43', '250.72', '250.53', '250.63', '250.73']
Diabetes uncomplicated	['E1[01234][019]']	['250[0123]']	['E11.9', 'E13.10', 'E13.9', 'E10.9', 'E11.00', 'E11.01', 'E10.10', 'E13.11']	['250.00', '250.02', '250.10', '250.12', '250.01', '250.13', '250.03', '250.20', '250.11', '250.32', '250.22', '250.0', '250.1', '250.30']
Drug abuse	['F1[12345689]', 'Z715', 'Z722']	['292', '304', '305[23456789]', 'V6542']	['F11.90', 'F11.23', 'F14.10', 'F12.10', 'F11.10', 'F19.90', 'F14.21', 'F19.188', 'F11.921', 'F19.20', 'F13.239', 'F11.20', 'F19.239', 'F13.20', 'F19.10', 'F12.20', 'F12.90', 'F11.220', 'F15.10', 'F19.21', 'Z71.51', 'F14.20', 'F14.129', 'F19.94', 'F19.982', 'F15.20', 'F19.950', 'F11.29', 'F19.129', 'F13.10', 'F15.982', 'F14.90', 'F11.11', 'F11.21', 'F13.231', 'F13.121', 'F15.23', 'F13.90', 'F11.24']	['305.60', '304.01', '305.20', '292.84', '292.81', '305.50', '305.62', '304.91', '304.20', '304.90', '304.00', '292.0', '305.63', 'V65.42', '305.93', '305.53', '304.03', '305.61', '305.90', '305.51', '304.23', '304.30', '305.40', '292.12', '305.23', '304.21', '305.71', '305.21', '292.9', '304.40', '304.13', '304.10', '304.61', '305.92', '305.91', '304.11', '292.89', '305.41', '305.73', '305.33', '304.31', '292.85', '305.22', '292.11', '304.93', '304.33', '305.43', '304.71', '305.52', '304.83', '305.70']
Fluid and electroylte disorders	['E222', 'E8[67]']	['2536', '276']	['E87.1', 'E87.0', 'E87.2', 'E87.6', 'E87.5', 'E86.0', 'E87.3', 'E22.2', 'E87.4', 'E87.70', 'E87.8', 'E86.1', 'E87.79', 'E87.71', 'E86.9']	['276.51', '276.7', '276.1', '276.52', '253.6', '276.8', '276.2', '276.3', '276.69', '276.50', '276.0', '276.61', '276.4', '276.5', '276.9', '276.6']
AIDS/HIV	['B2[0124]']	['04[234]']	['B20']	['042']

© 2019 Parikh RB et al. *JAMA Network Open.*

Hypertension, complicated	['I1[1235]']	['40[2345]']	['I12.9', 'I11.9', 'I12.0', 'I15.0', 'I15.1', 'I15.8', 'I15.9', 'I13.2', 'I13.0', 'I13.10', 'I11.0', 'I15.2']	['403.90', '402.90', '403.10', '404.90', '403.00', '404.91', '402.91', '403.11', '403.91', '402.11', '404.11', '402.10', '404.00', '405.19', '404.01', '405.01', '404.93', '404.10', '403.01', '405.99', '405.91', '404.03', '404.12', '402.00', '405.11', '404.92']
Hypertension, uncomplicated	['I10']	['401']	['I10']	['401.1', '401.9', '401.0', '401']
Hypothyroidism	['E0[0123]', 'E890']	['2409', '24([34]|6[18])']	['E03.9', 'E02', 'E03.8', 'E03.2', 'E89.0', 'E03.4', 'E01.0', 'E03.3', 'E03.1', 'E01.2']	['244.9', '240.9', '244.8', '246.8', '244.0', '244.1', '244.3', '246.1', '243', '244.2']
Liver disease	['B18', 'I8(5|64)', 'I982', 'K7(0|1[13457]|[234]|6[023456789])', 'Z944']	['070([23]|[45])', '456[012]', '57([01]|2[2345678]|3[3489])', 'V427']	['B18.2', 'K76.0', 'K74.60', 'K74.69', 'K76.9', 'K76.89', 'I85.10', 'K76.6', 'Z94.4', 'K70.30', 'B18.1', 'I85.00', 'K70.31', 'K73.9', 'K72.90', 'K74.0', 'K71.7', 'K72.00', 'K73.2', 'K70.9', 'B18.9', 'K70.10', 'I86.4', 'K74.3', 'K74.5', 'I85.01', 'I85.11', 'K76.7', 'K76.4', 'K71.10', 'B18.8', 'K76.5', 'K74.4', 'K76.3', 'K72.10', 'K70.40', 'K70.0', 'K70.11']	['573.3', '573.8', '573.9', '571.8', '456.1', '571.5', '070.54', '070.51', '571.41', '570', '070.30', '571.2', '571.3', '571.9', '572.3', '070.32', 'V42.7', '571.6', '571.49', '456.0', '456.20', '456.21', '573.4', '572.2', '070.2', '572.8', '070.44', '571.40', '571.42', '571.1', '571.0', '070.23', '070.33', '571.4', '070.59', '572.4', '571', '070.20']
Lymphoma	['C8[123458]', 'C96', 'C90[02]']	['20[012]', '2030', '2386']	['C83.00', 'C90.00', 'C83.80', 'C88.0', 'C85.99', 'C85.90', 'C85.10', 'C85.92', 'C82.90', 'C83.30', 'C85.80', 'C83.10', 'C81.00', 'C90.01', 'C84.70', 'C84.09', 'C84.69', 'C85.98', 'C83.31', 'C88.4', 'C85.93', 'C81.03', 'C85.83', 'C85.81', 'C85.89', 'C85.91', 'C83.01', 'C96.2', 'C81.90', 'C83.13', 'C81.98', 'C85.85', 'C84.00', 'C84.A0', 'C84.A8', 'C81.11', 'C82.41', 'C82.40', 'C90.02', 'C81.18', 'C82.20', 'C81.79', 'C81.71', 'C81.72', 'C81.12', 'C81.92', 'C90.20', 'C82.18', 'C82.94', 'C85.88', 'C83.98', 'C81.93', 'C81.40', 'C82.13', 'C90.21', 'C85.19', 'C85.82', 'C84.44', 'C82.38', 'C84.19', 'C81.15', 'C82.00', 'C85.94', 'C83.35', 'C82.97', 'C83.19', 'C85.97', 'C82.98', 'C84.49', 'C84.48', 'C85.87', 'C83.59', 'C85.16', 'C85.86', 'C83.39', 'C85.95', 'C83.32', 'C81.20', 'C81.19', 'C83.78', 'C83.79', 'C96.A', 'C83.34', 'C84.10', 'C82.91', 'C82.01', 'C82.16', 'C81.10', 'C81.99', 'C96.9', 'C85.13', 'C85.15', 'C82.10', 'C83.38', 'C85.18', 'C83.33', 'C83.37', 'C82.30', 'C81.94', 'C83.82', 'C82.11', 'C82.08', 'C83.50', 'C84.40', 'C83.51', 'C82.80', 'C81.01', 'C83.70', 'C83.73', 'C83.75', 'C81.91', 'C96.6', 'C83.08', 'C83.18', 'C81.74', 'C83.86',	['200.10', '202.80', '203.00', '200.80', '200.4', '200.40', '200.20', '200.30', '203.01', '238.6', '202.83', '202.03', '202.00', '202.88', '202.01', '200.31', '201.90', '200.70', '201.40', '200.60', '202.10', '200.7', '202.8', '202.81', '200.50', '200.51', '202.77', '200.3', '203.02', '202.60', '202.68', '202.05', '202.08', '200.11', '201.9', '201.91', '201.96', '203.0', '202.20', '200.71', '201.92', '201.98', '201.65', '202.85', '202.82', '202.87', '202.04', '200.77', '202.92', '202.84', '202.0', '201.93', '201.95', '201.50', '201.52', '200.33', '202.90', '200.00', '200.41', '202.40', '202.48', '200.76', '200.26', '200.75', '202.02', '200.34', '200.32', '200.62', '202.78', '202.86', '202.70', '200.01', '200.37', '200.48', '200.43', '200.73', '200.14', '202.07', '200.78', '200.08', '202.15', '201.60', '202.7', '200.13', '200.24', '200.28', '202.41', '201.68', '201.58', '202.13', '202.93', '202.06', '202.11', '201.10', '200.38', '200.1', '202.43', '200.61', '201.94', '201.20', '202.12', '202.18', '200.12', '202.50', '201.51', '200.42', '202.74', '200.74', '200.5', '200.88', '202.28', '200.72', '202.73', '200.21', '200.23', '200.44',

© 2019 Parikh RB et al. *JAMA Network Open.*

			'C82.81', 'C81.78', 'C82.26', 'C85.84', 'C85.12', 'C85.21', 'C85.20', 'C83.74', 'C96.Z', 'C83.09', 'C82.89', 'C82.09', 'C84.18', 'C82.95', 'C84.73', 'C84.72', 'C84.78', 'C83.07', 'C84.Z0', 'C82.61', 'C83.36', 'C83.89', 'C82.04', 'C84.41', 'C96.0', 'C85.26', 'C83.17', 'C81.13', 'C85.96', 'C85.22', 'C82.50', 'C82.06', 'C81.73', 'C82.99', 'C81.75', 'C82.23', 'C85.17', 'C83.15', 'C83.12', 'C81.70', 'C82.35', 'C82.93', 'C88.8', 'C81.08', 'C83.11', 'C82.60', 'C82.02', 'C85.11', 'C84.A9', 'C84.A4', 'C84.A6', 'C84.68', 'C84.60', 'C84.A1', 'C82.68', 'C84.45', 'C81.43', 'C84.43', 'C82.05', 'C82.69', 'C82.21', 'C83.58', 'C96.5', 'C83.76', 'C84.Z9', 'C84.42', 'C84.47', 'C84.07', 'C82.03', 'C83.71', 'C84.79', 'C85.28', 'C85.29', 'C85.14', 'C83.83', 'C84.A2', 'C82.84']	'200.47', '200.86', '200.35', '202.95', '202.47', '200.45', '202.30', '202.14', '200.63', '200.68', '202', '201.44', '202.4', '202.97', '202.58', '200.58', '201.61', '202.71', '200.46', '201.53', '201.64', '200.18', '202.98', '200.27', '200.36', '200.22', '201.02', '201.48', '200.2', '201.66', '202.36', '202.38', '201', '201.00', '202.96', '200.66', '200.65', '202.17', '202.75', '201.43', '201.97', '202.1', '202.31', '202.24', '201.54', '200.05', '202.91', '200.02', '201.05', '200.15', '200.64']
Metastatic cancer	['C7[789]', 'C80']	['19[6789]']	['C80.1', 'C79.51', 'C78.7', 'C78.01', 'C79.11', 'C77.0', 'C78.00', 'C79.70', 'C77.9', 'C79.9', 'C78.2', 'C79.60', 'C79.31', 'C77.8', 'C78.1', 'C78.6', 'C77.1', 'C79.89', 'C80.0', 'C78.02', 'C80.2', 'C79.2', 'C77.2', 'C78.4', 'C79.10', 'C79.82', 'C78.89', 'C79.49', 'C77.3', 'C77.4', 'C77.5', 'C79.00', 'C79.52', 'C78.39', 'C79.71', 'C79.01', 'C78.5', 'C79.19', 'C79.72', 'C79.02', 'C79.32', 'C79.61', 'C79.62', 'C79.81', 'C78.80', 'C78.30']	['196.3', '198.2', '198.3', '198.89', '199.1', '199', '199.0', '198.5', '196.0', '197.0', '196.1', '196.9', '198.7', '197.7', '198.6', '196.2', '197.4', '197.5', '197.6', '198.82', '198.81', '197.8', '196.6', '196.5', '198.1', '197.2', '198.4', '199.2', '196.8', '198.0', '197.3', '197.1', '196']
Obesity	['E66']	['2780']	['E66.9', 'E66.01', 'E66.3', 'E66.2', 'E66.8', 'E66.09']	['278.00', '278.01', '278.02', '278.03', '278.0']
Other neurological disorders	['G1[0123]', 'G2[012]', 'G25[45]', 'G31[289]', 'G3[2567]', 'G4[01]', 'G93[14]', 'R470', 'R56']	['3319', '332[01]', '333[45]', '33([45]|62)', '34([015]|8[13])', '78[04]3']	['R47.01', 'R47.02', 'R56.9', 'G31.84', 'G20', 'G40.909', 'G40.409', 'G40.109', 'G40.009', 'G35', 'G36.0', 'G13.0', 'G40.209', 'G11.9', 'G93.40', 'G40.301', 'G93.49', 'G40.802', 'G40.201', 'G31.83', 'G21.19', 'G40.219', 'G25.4', 'G93.41', 'G21.9', 'G37.9', 'G31.9', 'R56.1', 'G10', 'G12.21', 'G40.89', 'G40.119', 'G40.309', 'G40.801', 'G40.019', 'G40.B09', 'G40.A09', 'G40.001', 'G40.901', 'G37.3', 'G25.5', 'G11.3', 'G40.311', 'G40.319', 'G31.89', 'G11.8', 'G32.81', 'G31.85', 'G93.1', 'G40.111', 'G31.2', 'G40.B19', 'G40.419', 'G40.211', 'G40.911', 'G12.20', 'G40.804', 'G40.401', 'G40.501', 'G13.1', 'G11.4']	['345.90', '345.80', '780.39', '334.4', '345.40', '332.0', '784.3', '348.39', '780.31', '348.30', '345.3', '345.10', '345.50', '345.91', '340', '341.0', '348.31', '345.00', '345.41', '335.10', '334.9', '334.3', '341.9', '348.1', '345.2', '331.9', '341.2', '332.1', '334.2', '333.5', '335.21', '335.20', '780.33', '333.4', '345.51', '345.71', '345.70', '341.8', '345.11', '334.0', '345.01', '334.1']

© 2019 Parikh RB et al. *JAMA Network Open.*

Pulmonary circulation disorder	['I2([67]|8[089])']	['415[01]', '416', '417[089]']	['I27.2', 'I27.0', 'I26.09', 'I26.99', 'I27.9', 'I27.82', 'I26.90', 'I28.8', 'I27.81', 'I26.02', 'I27.20', 'I27.89', 'I27.21', 'I27.29', 'I26.92', 'I28.0']	['416.9', '416.8', '416.0', '415.19', '416.2', '415.11', '415.13', '415.0', '417.8', '417.0', '415.12', '417.9']
Peptic ulcer disease excluding bleeding	['K2[5678][79]']	['53[1234][79]']	['K25.9', 'K26.9', 'K27.9', 'K25.7', 'K28.9']	['533.90', '531.90', '532.90', '531.70', '534.90', '531.71', '532.70', '531.91', '534.70', '534.91', '533.70', '532.91']
Pulmonary valvular disorder	['I7([01]|3[189]|71|9[02])', 'K55[189]', 'Z95[89]']	['0930', '4373', '44([01]|3[123456789])', '4471', '557[19]', 'V434']	['Z95.810', 'I70.0', 'I73.9', 'I71.4', 'I71.2', 'I70.209', 'Z95.820', 'Z95.828', 'I71.6', 'I70.219', 'I70.212', 'I70.92', 'Z95.818', 'Z95.9', 'I70.312', 'I77.1', 'I70.8', 'I71.3', 'I71.9', 'I70.261', 'I71.01', 'K55.9', 'I70.201', 'K55.8', 'Z95.811', 'I70.262', 'I71.1', 'I70.90', 'I70.203', 'I70.1', 'I70.213', 'I70.211', 'K55.1', 'I70.245', 'I71.03', 'I70.91', 'I71.02', 'I71.00', 'I70.293', 'I70.25', 'I71.5', 'I70.235', 'I70.361', 'I73.89', 'I70.221', 'I73.1']	['441.2', '437.3', '440.9', '440.23', '443.9', '557.9', '440.0', '440.21', '441.4', '440.20', '447.1', '443.89', '440.31', '443.21', '557.1', '441.00', '441.7', '440.4', '441.9', '440.8', '440.24', '440.30', '440.1', '440.22', '441.01', '441.02', '441.3', '443.81', '443.24', '443.82', '443.29', '443.22', '441.03', '440.29', 'V43.4', '441.1', '440.32', '443.1']
Paralysis	['G041', 'G114', 'G8(0[12]|[12]|3[012349])']	['3341', '34([23]|4[01234569])']	['G83.4', 'G82.52', 'G81.91', 'G81.90', 'G83.0', 'G81.94', 'G82.22', 'G82.20', 'G83.9', 'G83.11', 'G80.1', 'G83.10', 'G81.14', 'G82.50', 'G82.21', 'G81.93', 'G81.92', 'G11.4']	['344.60', '342.92', '343.9', '342.90', '344.04', '344.9', '344.2', '344.61', '344.5', '342.10', '342.91', '344.1', '344.30', '342.12', '342.01', '344.00', '342.02', '344.40', '343.8', '343.0', '342.80', '342.82', '343.1', '342.00', '334.1']
Psychoses	['F2[0234589]', 'F3([01]2|15)']	['2938', '296[0145]4', '29[578]']	['F20.9', 'F25.9', 'F20.0', 'F20.3', 'F24', 'F20.1', 'F20.89', 'F20.81', 'F23', 'F25.0', 'F22', 'F31.5', 'F29', 'F28']	['293.83', '297.1', '297.9', '298.9', '295.70', '295.90', '295.72', '295.30', '295.60', '295.62', '295.00', '295.74', '295.45', '295.95', '293.84', '295.32', '297.3', '298.8', '295.92', '295.10', '295.42', '296.44', '298.1', '293.82', '295.40', '298.2', '295.80', '295.34', '293.89', '298.3', '295.50', '296.54', '295.94']
Renal failure	['I120', 'I131', 'N1[89]', 'N250', 'Z49[012]', 'Z940', 'Z992']	['403', '404', '58([56]|80)', 'V4(20|51)', 'V56']	['N18.2', 'N18.9', 'N18.3', 'N18.1', 'N18.4', 'I12.0', 'N18.6', 'Z99.2', 'N19', 'N18.5', 'Z94.0', 'N25.0', 'Z49.01', 'I13.10']	['403.90', '585.9', '585.3', '585.5', '403.10', '404.90', '585.4', '403.00', '404.91', '585.2', '403.11', '585', '403.91', '585.6', 'V45.11', '585.1', '586', 'V42.0', '404.11', '588.0', 'V45.1', 'V56.1', 'V56.2', 'V45.12', '404.00', '404.01', 'V56.0', '404.93', '404.10', '403.01', '404.03', '404.12', '404.92', 'V56.8']
Rheumatoid arthritis/collagen vascular diseases	['L94[013]', 'M0[568]', 'M12[03]', 'M3(0|1[0123]|[2345])', 'M4(5|6[189])']	['446', '7010', '71(0[0123489]|12|4|93)', '72([05]|8[58]|93)']	['M06.9', 'M32.9', 'M35.8', 'M35.9', 'M35.00', 'M35.3', 'M46.87', 'M33.90', 'M46.1', 'M06.4', 'M46.92', 'M33.20', 'M32.10', 'M32.14', 'M31.1', 'M32.13', 'M31.30', 'M06.011', 'M32.19', 'M34.1', 'M46.86', 'M35.1', 'M35.2', 'M34.9', 'M06.851', 'M06.852', 'M46.96', 'M45.9', 'L94.0', 'M35.01', 'M06.00', 'M34.89', 'M06.09', 'M31.0', 'M34.81', 'M33.22',	['728.85', '714.0', '720.2', '728.88', '728.87', '446.5', '710.0', '710.9', '710.2', '701.0', '710.8', '725', '728.89', '728.84', '714.9', '710.3', '719.32', '714.81', '720.9', '714.89', '720.0', '729.39', '710.4', '728.83', '710.1', '728.86', '446.4', '714.30', '446.6', '446.0', '729.30', '728.5', '714.1', '446.20', '446.7', '728.82',

© 2019 Parikh RB et al. *JAMA Network Open.*

			'M46.82', 'M30.0', 'M35.09', 'M08.00', 'M05.9', 'M33.92', 'M46.90', 'M05.79', 'M05.871', 'M35.7', 'M31.31', 'M30.1', 'M06.1', 'M06.30', 'M30.3', 'M08.3']	'719.30', '714.2', '720.81', '446.29', '720.89', '446.1', '720.1']
Solid tumor without metastasis	['C[01]', 'C2[0123456]', 'C3[01234789]', 'C4[01356789]', 'C5[012345678]', 'C6', 'C7[0123456]', 'C97']	['1[456]', '17[012456789]', '18', '19([012345])']	['C34.90', 'C34.12', 'C34.92', 'C50.912', 'C50.911', 'C50.919', 'C43.70', 'C43.60', 'C43.0', 'C25.0', 'C25.9', 'C64.9', 'C61', 'C34.30', 'C18.9', 'C43.9', 'C34.81', 'C54.1', 'C54.9', 'C07', 'C15.9', 'C67.2', 'C67.9', 'C34.2', 'C34.31', 'C34.00', 'C34.91', 'C34.11', 'C16.9', 'C49.9', 'C17.9', 'C26.0', 'C34.32', 'C23', 'C67.0', 'C67.8', 'C22.1', 'C20', 'C46.0', 'C55', 'C56.9', 'C22.0', 'C73', 'C50.412', 'C24.1', 'C34.02', 'C50.112', 'C34.10', 'C32.9', 'C64.2', 'C18.1', 'C18.0', 'C25.8', 'C08.9', 'C19', 'C45.0', 'C21.0', 'C21.1', 'C76.3', 'C50.212', 'C22.8', 'C43.4', 'C51.9', 'C50.812', 'C71.9', 'C50.811', 'C52', 'C24.9', 'C45.9', 'C41.0', 'C76.0', 'C62.91', 'C63.7', 'C32.1', 'C34.01', 'C22.9', 'C71.2', 'C46.9', 'C41.1', 'C50.419', 'C49.6', 'C49.0', 'C30.1', 'C50.511', 'C71.1', 'C54.0', 'C18.5', 'C18.4', 'C50.819', 'C56.2', 'C25.3', 'C43.59', 'C67.4', 'C62.90', 'C37', 'C66.9', 'C06.9', 'C47.3', 'C06.2', 'C47.9', 'C01', 'C53.9', 'C50.211', 'C22.7', 'C68.8', 'C68.0', 'C25.7', 'C67.5', 'C16.0', 'C43.71', 'C64.1', 'C65.9', 'C15.5', 'C15.3', 'C41.9', 'C40.20', 'C49.12', 'C76.42', 'C69.12', 'C49.22', 'C49.20', 'C10.9', 'C14.0', 'C26.9', 'C31.9', 'C11.9', 'C30.0', 'C17.0', 'C50.611', 'C50.219', 'C50.411', 'C34.80', 'C57.00', 'C50.312', 'C25.4', 'C43.31', 'C04.9', 'C25.2', 'C02.9', 'C26.1', 'C49.5', 'C17.2', 'C47.5', 'C47.0', 'C72.0', 'C10.4', 'C18.7', 'C68.9', 'C43.30', 'C53.0', 'C18.2', 'C50.921', 'C57.02', 'C09.0', 'C50.612', 'C25.1', 'C21.8', 'C50.311', 'C18.6', 'C69.00', 'C69.01', 'C75.9', 'C09.9', 'C71.8', 'C71.4', 'C31.0', 'C05.0', 'C48.0', 'C71.3', 'C71.6', 'C74.91', 'C49.3', 'C50.822', 'C41.3', 'C56.1', 'C74.90', 'C62.92', 'C62.12', 'C03.9', 'C16.1', 'C02.1', 'C11.2', 'C69.92', 'C69.22', 'C69.90', 'C69.32', 'C76.2', 'C43.62', 'C72.9', 'C02.0', 'C76.1', 'C50.011', 'C34.82', 'C50.319', 'C57.9', 'C17.1', 'C48.1',	['174.9', '174.1', '174.3', '171.4', '174.4', '172.0', '172.6', '172.5', '188.9', '172.9', '172.7', '189.0', '185', '162.3', '162.5', '162.9', '153.2', '153.9', '153.3', '171.5', '171.9', '174.2', '174.0', '182.0', '184.9', '150.4', '150.9', '174.8', '152.9', '183.0', '152.1', '193', '172.2', '188.8', '156.0', '188.0', '188.4', '155.1', '154.1', '176.0', '179', '183.8', '162.8', '151.9', '190.1', '174.5', '156.2', '183', '190.3', '153.8', '161.9', '161.0', '162.2', '182.1', '163.9', '157.0', '157.9', '174.6', '142.9', '142.1', '153.0', '153.1', '150.8', '150.0', '153.6', '155.0', '154.0', '154.3', '172.4', '184.4', '153.4', '171.2', '171.0', '155.2', '188.2', '151.0', '191.9', '191.1', '192.9', '170.0', '186.9', '187.9', '187.8', '187.7', '158.9', '161.1', '195.0', '191.2', '176.9', '183.3', '170.1', '145.9', '145.6', '143.1', '180.8', '151.3', '175.9', '154.2', '157.3', '164.9', '164.2', '189.9', '174', '164.0', '149.0', '180.9', '189.2', '189.1', '156.1', '146.0', '146.9', '150.5', '147.9', '141.0', '141.9', '180.1', '180.0', '151.8', '162.4', '188.1', '151.4', '189.3', '184.0', '159.9', '157.8', '171.3', '190.6', '152.0', '156.9', '159.8', '171.6', '153.5', '192.2', '158.0', '183.2', '154.8', '190.2', '190.7', '142.0', '147.0', '157.4', '172.3', '170.4', '176.4', '156.8', '170.9', '144.9', '195.8', '152.2', '194.0', '170.2', '195.5', '190.5', '189.8', '159.1', '153.7', '142.8', '158.8', '157.2', '146.1', '144.8', '149.8', '148.1', '195.1', '150.2', '195.2', '194.9', '183.9', '176.8', '160.2', '145.2', '147.8', '145.0', '191.3', '191.8', '191.6', '191.7', '141.1', '141.2', '157.1', '170.3', '143.9', '143.8', '192.1', '145.3', '147.1', '141.6', '171.8', '190.9', '160.9', '160.0', '188.6', '172.8', '148.8', '161.3', '141.8', '170.7', '145.4', '195', '195.3', '194.3', '146.2', '183.4', '145.8', '144.0', '140.8', '140.0', '140.9', '149.9', '141.3', '146.8', '184', '188.7', '163.0', '163.1', '188.3', '176.2', '172', '172.1', '184.1', '191.0', '151.2', '171.7', '151.1', '146.3', '186.0', '188.5', '195.4', '175.0', '182',

© 2019 Parikh RB et al. *JAMA Network Open.*

			'C48.2', 'C65.2', 'C66.2', 'C50.519', 'C70.0', 'C62.10', 'C05.1', 'C57.01', 'C50.512', 'C49.21', 'C02.3', 'C06.0', 'C04.0', 'C06.80', 'C57.4', 'C70.9', 'C67.7', 'C32.0', 'C43.61', 'C49.A2', 'C54.8', 'C65.1', 'C74.92', 'C50.111', 'C43.72', 'C38.4', 'C16.2', 'C43.39', 'C40.01', 'C40.00', 'C40.02', 'C50.929', 'C50.922', 'C50.021', 'C70.1', 'C33', 'C76.52', 'C15.8', 'C31.1', 'C43.10', 'C69.91', 'C69.30', 'C67.1', 'C71.0', 'C15.4', 'C53.8', 'C46.4', 'C75.5', 'C75.2', 'C58', 'C43.20', 'C74.00', 'C49.A0', 'C49.A4', 'C18.3', 'C49.4', 'C13.9', 'C60.1', 'C60.9', 'C71.7', 'C10.0', 'C31.3', 'C31.2', 'C31.8', 'C16.3', 'C67.3', 'C69.21', 'C51.0', 'C16.5', 'C38.1', 'C49.10', 'C75.1', 'C14.8', 'C54.2', 'C12', 'C49.11', 'C53.1', 'C43.21', 'C75.0', 'C74.10', 'C74.11', 'C22.3', 'C24.0', 'C05.2', 'C69.61', 'C13.0', 'C63.11', 'C50.022', 'C40.21', 'C38.2', 'C67.6', 'C13.1', 'C32.8', 'C09.1', 'C76.8', 'C50.619', 'C41.2', 'C41.4', 'C63.8', 'C16.4', 'C09.8', 'C16.8', 'C48.8', 'C46.1', 'C50.019', 'C38.3', 'C16.6', 'C13.8', 'C04.1', 'C04.8', 'C05.9', 'C08.0', 'C69.52', 'C38.8', 'C10.3', 'C11.1', 'C40.22', 'C45.7', 'C45.1', 'C43.8', 'C50.121', 'C50.119', 'C43.12', 'C69.42', 'C71.5', 'C50.012', 'C43.52', 'C02.4', 'C74.02', 'C43.22', 'C03.0', 'C66.1', 'C76.51', 'C69.51', 'C69.50', 'C00.0', 'C10.8', 'C74.12', 'C22.2', 'C72.59', 'C32.3', 'C69.10', 'C32.2', 'C11.8', 'C11.3', 'C54.3', 'C69.62', 'C51.8', 'C10.2', 'C49.8', 'C62.11', 'C10.1', 'C69.60', 'C69.11', 'C47.22', 'C72.32', 'C22.4', 'C03.1', 'C40.11', 'C68.1', 'C69.41', 'C39.9']	'191.4', '151.6', '161.8', '162', '159.0', '191.5', '194.6', '171', '162.0', '148.9', '190.4', '151.5', '182.8', '164.1', '161.2', '140.4', '140.1', '147.2', '146.4', '160.5', '141', '150.3', '150.1', '184.8', '160.8', '170.6', '194.1', '148.0', '148.2', '170.8', '192.0', '160.3', '146', '187.2', '187.4', '176.3', '194.8', '176.1', '144.1', '145.5', '141.4', '160.4', '146.7', '153', '161', '189', '164.3', '194.5', '143.0', '170.5', '152', '164.8', '163.8', '146.6', '141.5', '148.3', '145.1', '146.5', '152.8']
Valvular disease	['A520', 'I0[5678]', 'I09[18]', 'I3[456789]', 'Q23[0123]', 'Z95[234]']	['0932', '39[4567]', '424', '746[3456]', 'V422', 'V433']	['I05.9', 'I34.0', 'Z95.4', 'I35.1', 'I35.9', 'Z95.2', 'I38', 'I08.3', 'I35.0', 'I07.1', 'I34.1', 'I35.8', 'I08.0', 'I08.2', 'I36.1', 'Z95.3', 'I35.2', 'I05.0', 'I08.1', 'I05.2', 'I06.1', 'I06.0', 'Q23.1', 'I09.81', 'I37.0', 'I06.2', 'Q23.0', 'I07.9', 'I05.1', 'I34.8', 'I36.9', 'I37.1', 'I34.2']	['424.0', '424.1', '397.0', '394.9', '424.90', '395.0', 'V43.3', '396.3', 'V42.2', '746.6', '396.2', '424.2', '396.8', '746.4', '394.2', '394.1', '396.9', '394.0', '395.1', '746.3', '396.0', '424.91', '424.3', '396.1', '746.5', '395.2', '395.9', '397.9', '424.99', '424']
Weight loss	['E4[0123456]', 'R634', 'R64']	['26[0123]', '7832', '7994']	['R63.4', 'E44.0', 'E43', 'E44.1', 'E46', 'R64', 'E40', 'E41']	['783.21', '263.9', '261', '262', '799.4', '263.0', '263.1', '783.22', '263.8', '260']

© 2019 Parikh RB et al. *JAMA Network Open.*

II. Stepwise logistic regression output

Logit Regression Results

Dep. Variable:	label	No. Observations:	18567
Model:	Logit	Df Residuals:	18532
Method:	MLE	Df Model:	34
Date:	Thu, 22 Aug 2019	Pseudo R-squ.:	0.2369
Time:	11:50:07	Log-Likelihood:	-2312.0
converged:	True	LL-Null:	-3029.7
Covariance Type:	nonrobust	LLR p-value:	4.679e-280

	coef	std err	z	P>|z|	[0.025	0.975]
intercept	-3.9880	0.061	-65.132	0.000	-4.108	-3.868
MCHC: last	-0.1918	0.041	-4.720	0.000	-0.271	-0.112
Platelets: last	-0.0584	0.035	-1.656	0.098	-0.127	0.011
Red Blood Cells: last	-0.2117	0.042	-5.005	0.000	-0.295	-0.129
Calcium: last	0.1127	0.035	3.204	0.001	0.044	0.182
Alkaline Phosphatase: last	0.1147	0.020	5.622	0.000	0.075	0.155
Albumin: last	-0.3849	0.038	-10.250	0.000	-0.458	-0.311
Q-T Interval: last	-0.0889	0.029	-3.072	0.002	-0.146	-0.032
FERRITIN: last	0.0499	0.021	2.400	0.016	0.009	0.091
IMMUNOGLOBULINS A: last	0.0563	0.024	2.373	0.018	0.010	0.103
RBC: last	-0.1804	0.036	-4.975	0.000	-0.251	-0.109
BUN: last	0.1114	0.025	4.509	0.000	0.063	0.160
Beta-Globulin Fracti: last	-0.0683	0.033	-2.071	0.038	-0.133	-0.004
n_METS_recent	0.2247	0.026	8.525	0.000	0.173	0.276
n_WL_recent	0.0682	0.024	2.819	0.005	0.021	0.116
n_DA_recent	-0.0929	0.053	-1.745	0.081	-0.197	0.011
n_Lymp	-0.2457	0.102	-2.420	0.016	-0.445	-0.047
n_Lymp_recent	0.2746	0.080	3.419	0.001	0.117	0.432
n_PCD_recent	0.0825	0.028	2.918	0.004	0.027	0.138
n_Obesity_recent	-0.0825	0.047	-1.757	0.079	-0.175	0.010
n_HPTN_UC_recent	-0.1040	0.041	-2.519	0.012	-0.185	-0.023
n_Fluid_recent	0.1046	0.026	3.966	0.000	0.053	0.156
n_OthND_recent	0.1050	0.028	3.794	0.000	0.051	0.159
n_PVD	0.0635	0.032	2.004	0.045	0.001	0.126
n_VD_recent	-0.0899	0.043	-2.114	0.035	-0.173	-0.007

© 2019 Parikh RB et al. *JAMA Network Open.*

n_Drug	0.0944	0.030	3.124	0.002	0.035	0.154
n_Diab_C	0.1082	0.044	2.486	0.013	0.023	0.194
n_Diab_C_recent	-0.1011	0.048	-2.106	0.035	-0.195	-0.007
n_Coag_recent	0.0739	0.029	2.509	0.012	0.016	0.132
n_BLA	0.2588	0.051	5.027	0.000	0.158	0.360
n_BLA_recent	-0.2777	0.077	-3.613	0.000	-0.428	-0.127
n_Tumor	-0.4069	0.080	-5.060	0.000	-0.564	-0.249
n_Tumor_recent	0.7200	0.072	10.022	0.000	0.579	0.861
PAT_AGE	0.4277	0.051	8.389	0.000	0.328	0.528
SEX_C	0.2308	0.042	5.548	0.000	0.149	0.312

© 2019 Parikh RB et al. *JAMA Network Open.*

III. Feature Selection

1. Remove variables with zero variance (766 → 751 features)

```
var = train[in_vars].var()
zero_variance_vars = list(var[var == 0.0].index)
zero_variance_vars
# Drop zero variance features
in_vars = [v for v in in_vars if v not in zero_variance_vars]
```

2. Group highly inter-correlated features (751 → 590 features)

```
# drop correlated features, retain a map of redundancy
CORRELATION_THRESHOLD = 0.95

grouped = []
grouped_dict = {}
cor_map = train[in_vars].corr()
for var in cor_map.index:
    if var not in grouped:
        cor_vec = cor_map.loc[var,:]
        idb_cor =  cor_vec > CORRELATION_THRESHOLD
        collected = list(cor_vec[idb_cor].index)
        collected = [c for c in collected if c != var]
        grouped.extend(collected)
        grouped_dict.update({var: collected})
# Drop those variables that have been grouped into a single representative
in_vars = [v for v in in_vars if v not in grouped]
```

3. Dropping variables with no mutual optimization (590 → 559 features)

```
from sklearn.feature_selection import SelectKBest
from sklearn.feature_selection import mutual_info_classif
bestfeatures = SelectKBest(score_func=mutual_info_classif, k=10)
fit = bestfeatures.fit(train[in_vars], train['label'])
dfscores = pd.DataFrame(fit.scores_)
dfcolumns = pd.DataFrame(train[in_vars].columns)
#concat two dataframes for better visualization
featureScores = pd.concat([dfcolumns,dfscores],axis=1)
featureScores.columns = ['var','score']
print(featureScores.nlargest(10,'score'))
#drop those with no mutual information
zero_mutual_info_vars = featureScores[featureScores['score'] == 0.0]['var'
].values
zero_mutual_info_vars
in_vars = [v for v in in_vars if v not in zero_mutual_info_vars]
```

© 2019 Parikh RB et al. *JAMA Network Open.*

IV. Hyperparameters for random forest and gradient boosting algorithms

Random Forest

```
RandomForestClassifier(bootstrap=True, compute_importances=None,
    criterion='entropy', max_depth=None, max_features='auto',
    max_leaf_nodes=None, min_density=None, min_samples_leaf=1,
    min_samples_split=2, n_estimators=200, n_jobs=4,
    oob_score=False, random_state=None, verbose=0)
```

Gradient Boosting

```
GradientBoostingClassifier(criterion='friedman_mse', init=None,
        learning_rate=0.01, loss='deviance', max_depth=10,
        max_features='sqrt', max_leaf_nodes=None,
        min_impurity_decrease=0.0, min_impurity_split=None,
        min_samples_leaf=4, min_samples_split=2,
        min_weight_fraction_leaf=0.0, n_estimators=890,
        n_iter_no_change=5, presort='auto', random_state=42,
        subsample=1.0, tol=0.0001, validation_fraction=0.1,
        verbose=1, warm_start=False)
```

Hyperparameter tuning of the Random Forest and Gradient Boosting models was conducted by random grid search over 500 iterations using k=5-fold cross-validation for a total of 50,000 model fits. The scoring function used was RoC-AUC. The hyper-parameter space searched over and optimal values are presented in the following table.

Hyperparameter search space

Model	Hyperparameter	Description	Search Space	Optimal
Random Forest	n_estimators	The number of trees in the forest.	[10, 120, 230, 340, 450, 560, 670, 780, 890, 1000]	340
Random Forest	max_features	The number of features to consider when looking for the best split.	['auto', 'sqrt']	‘auto’
Random Forest	max_depth	Maximum depth of the individual regression estimators.	[5, 10, 15, 20, 25, 30, 35, 40, 45, 50, 55, 60, 65, 70, 75, 80, 85, 90, 95, 100, None]	45
Random Forest	min_samples_split	The minimum number of samples required to split an internal node	[2, 5, 10]	5
Random Forest	min_samples_leaf	The minimum number of samples required to be at a leaf node.	[1, 2, 4]	4

© 2019 Parikh RB et al. *JAMA Network Open.*

Gradient Boosting	max_depth	Maximum depth of the individual regression estimators.	[5, 10, 15, 20, 25, 30, 35, 40, 45, 50, 55, 60, 65, 70, 75, 80, 85, 90, 95, 100, None]	10
Gradient Boosting	max_features	The number of features to consider when looking for the best split	['auto', 'sqrt']	‘sqrt’
Gradient Boosting	min_samples_leaf	The minimum number of samples required to be at a leaf node.	[1, 2, 4]	4
Gradient Boosting	min_samples_split	The minimum number of samples required to split an internal node	[2, 5, 10]	2
Gradient Boosting	n_estimators	The number of boosting stages to perform.	[10, 120, 230, 340, 450, 560, 670, 780, 890, 1000]	890
Gradient Boosting	subsample	The fraction of samples to be used for fitting the individual base learners.	[0.5, 0.8, 1.0]	1.0
Gradient Boosting	learning_rate	The learning rate shrinks the contribution of each tree by that value.	[0.01, 0.03111111, 0.05222222, 0.07333333, 0.09444444, 0.11555556, 0.13666667, 0.15777778, 0.17888889, 0.2]	0.01
Gradient Boosting	loss	The loss function to be optimized.	['deviance', 'exponential']	'deviance'

© 2019 Parikh RB et al. *JAMA Network Open.*

eReferences.

1. Elfiky AA, Pany MJ, Parikh RB, Obermeyer Z. Development and Application of a Machine Learning Approach to Assess Short-term Mortality Risk Among Patients With Cancer Starting Chemotherapy. *JAMA Netw Open*. 2018;1(3):e180926-e180926. doi:10.1001/jamanetworkopen.2018.0926

2. Bertsimas D, Dunn J, Pawlowski C, et al. Applied Informatics Decision Support Tool for Mortality Predictions in Patients With Cancer. *JCO Clinical Cancer Informatics*. June 2018. doi:10.1200/CCI.18.00003

3. Gupta S, Tran T, Luo W, et al. Machine-learning prediction of cancer survival: a retrospective study using electronic administrative records and a cancer registry. *BMJ Open*. 2014;4(3). doi:10.1136/bmjopen-2013-004007

4. Sahni N, Simon G, Arora R. Development and Validation of Machine Learning Models for Prediction of 1-Year Mortality Utilizing Electronic Medical Record Data Available at the End of Hospitalization in Multicondition Patients: a Proof-of-Concept Study. *J GEN INTERN MED*. 2018;33(6):921-928. doi:10.1007/s11606-018-4316-y

5. Weng SF, Reps J, Kai J, Garibaldi JM, Qureshi N. Can machine-learning improve cardiovascular risk prediction using routine clinical data? *PLoS ONE*. 2017;12(4):e0174944. doi:10.1371/journal.pone.0174944

6. Churpek MM, Yuen TC, Winslow C, Meltzer DO, Kattan MW, Edelson DP. Multicenter Comparison of Machine Learning Methods and Conventional Regression for Predicting Clinical Deterioration on the Wards. *Critical care medicine*. 2016;44(2):368. doi:10.1097/CCM.0000000000001571

7. Taylor RA, Pare JR, Venkatesh AK, et al. Prediction of In-hospital Mortality in Emergency Department Patients With Sepsis: A Local Big Data–Driven, Machine Learning Approach. *Academic emergency medicine : official journal of the Society for Academic Emergency Medicine*. 2016;23(3):269. doi:10.1111/acem.12876

© 2019 Parikh RB et al. *JAMA Network Open.*

eFigure 1. Cohort Selection Process

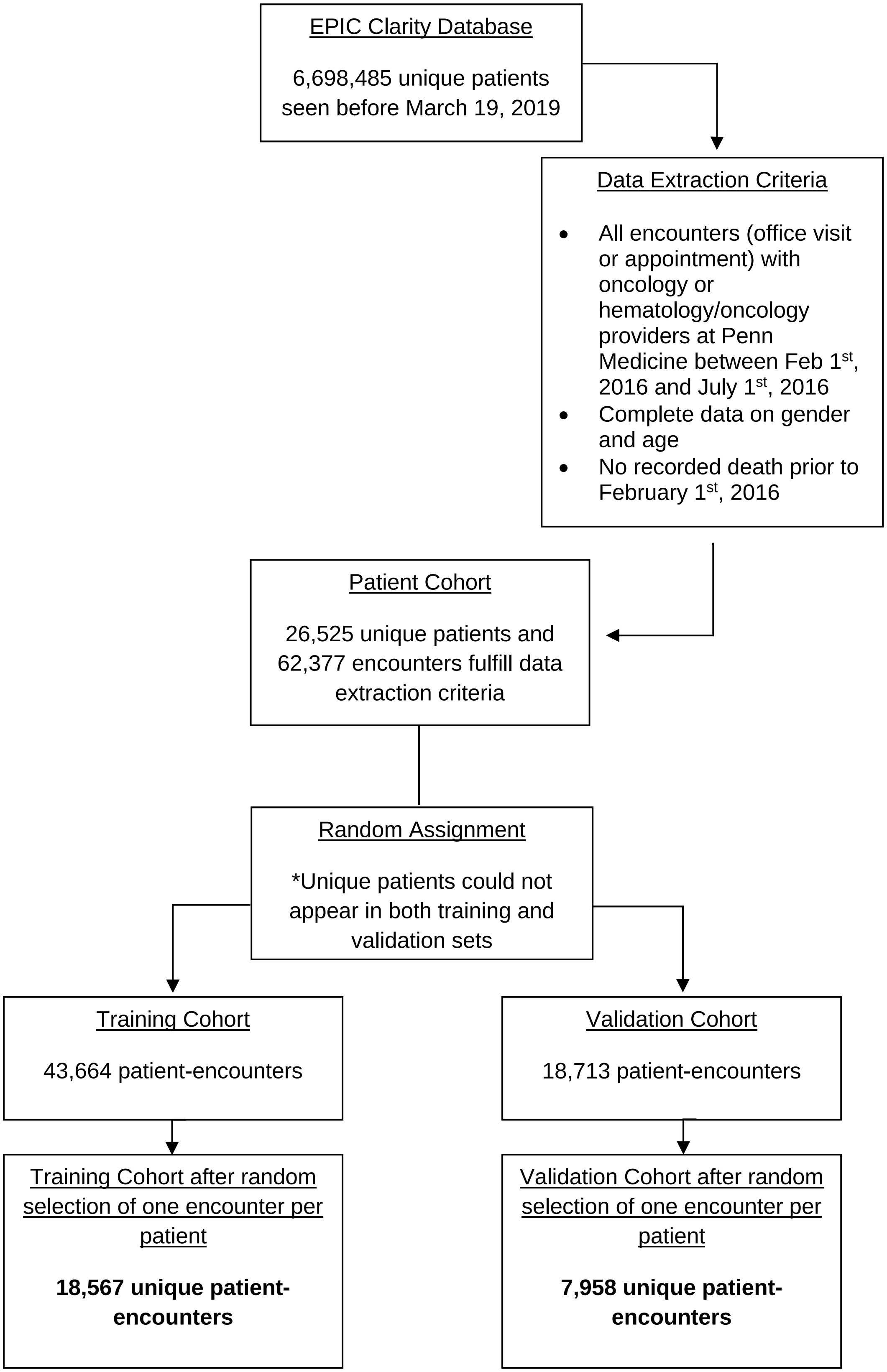

© 2019 Parikh RB et al. *JAMA Network Open.*

eFigure 2. Model Calibration Plots

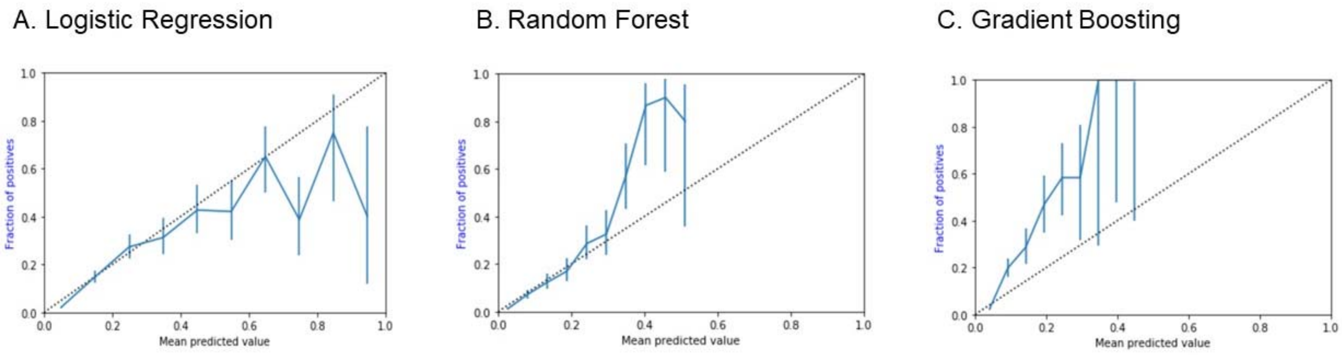

Footnote: Calibration plots describing observed (y-axis) vs. predicted (x-axis) mortality for the logistic regression (A), random forest (B), and gradient boosting (C) models. Each point represents one of ten bins of predicted probability of 180-day mortality for each model. The observed rate of 180-day mortality for each of the probability bins is plotted on the y-axis. The 45-degree dotted diagonal line represents points along a perfectly-calibrated model. The bars show 95% confidence intervals around the observed probability.

© 2019 Parikh RB et al. *JAMA Network Open.*

eFigure 3. Observed 180-Day Survival by Varying Risk Thresholds for Random Forest Model

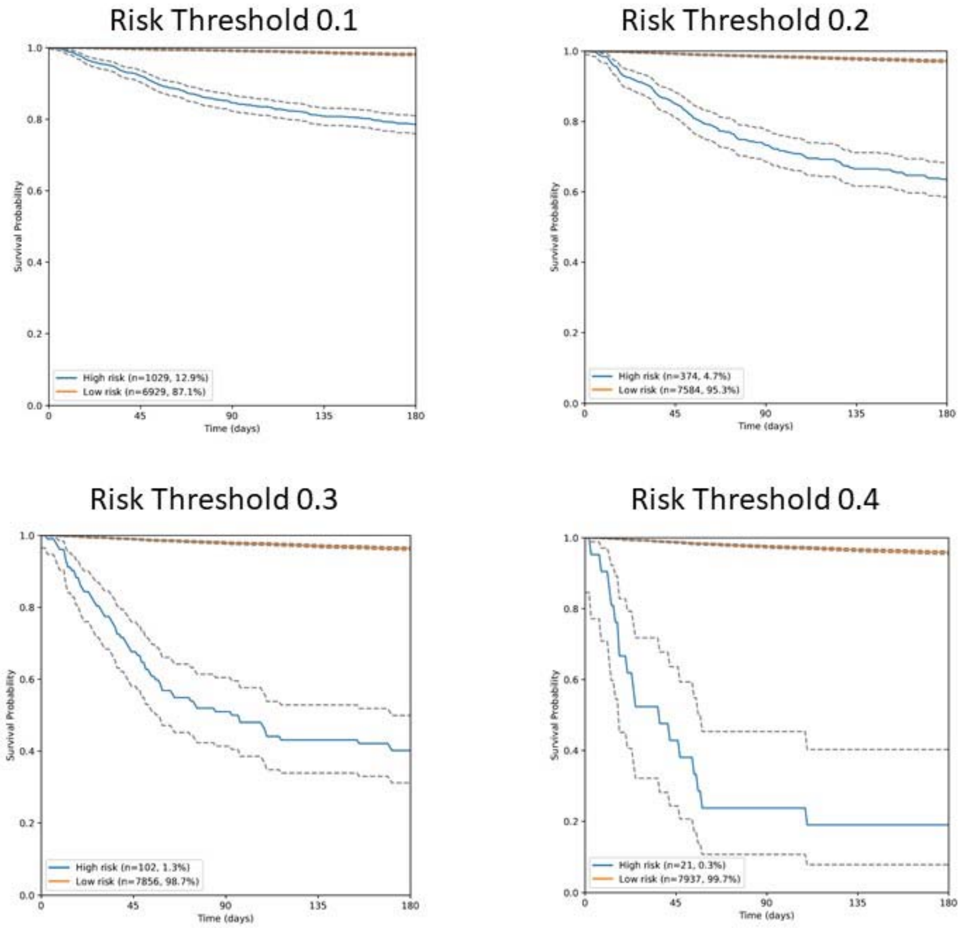

Footnote: Risk threshold refers to proportion risk of 180-day mortality, as predicted by the random forest algorithm (e.g. 0.2 = 20% predicted risk). Solid lines refer to observed survival; orange lines refer to patients falling under the risk threshold, whereas blue lines refer to patients falling above the risk threshold. Dotted lines refer to 95% confidence intervals.

© 2019 Parikh RB et al. *JAMA Network Open.*

eFigure 4. Observed 500-Day Survival by Predicted Risk for Random Forest Model

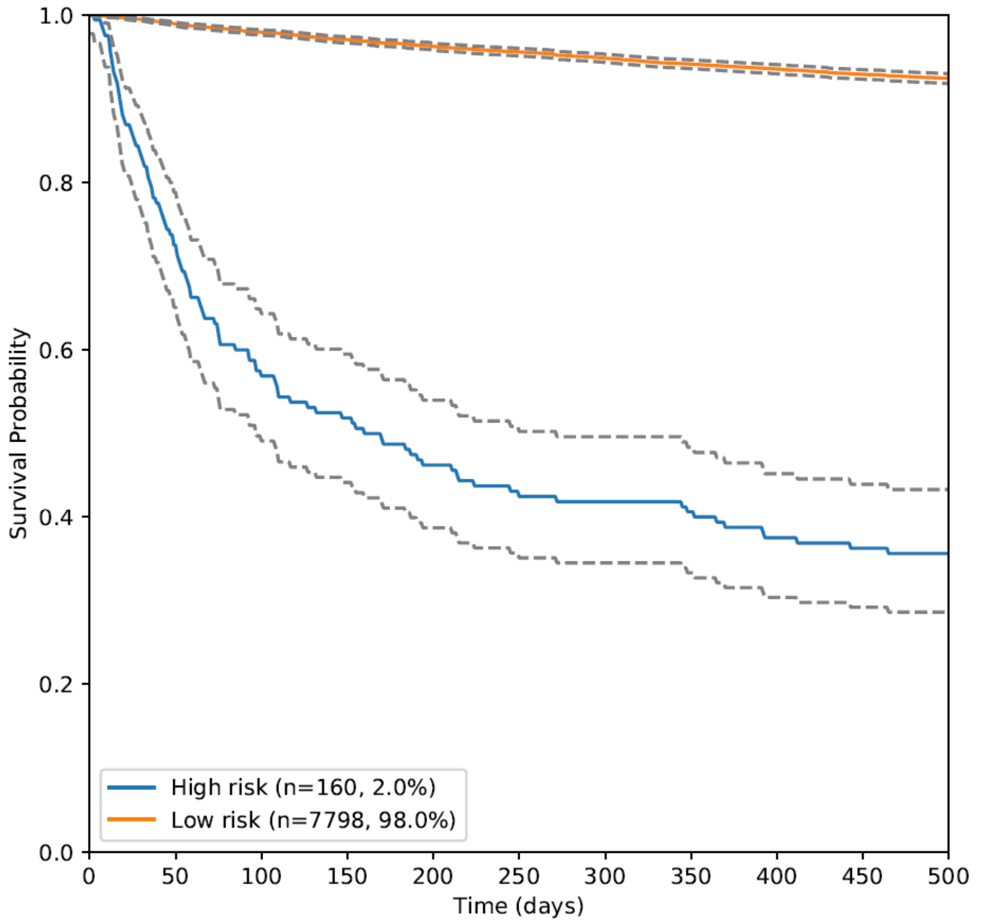

High Risk n Survived	160	116	91	83	74	68	67	65	60	58	57
Low Risk n Survived	7798	7712	7635	7567	7504	7451	7393	7338	7293	7243	7206

Footnote: Risk threshold was determined in the random forest model by setting the alert rate to 0.02, which corresponds to a proportion risk of 180-day mortality of 27%. Solid lines refer to observed survival; orange lines refer to patients falling under the risk threshold, whereas blue lines refer to patients falling above the risk threshold. Dotted lines refer to 95% confidence intervals.

© 2019 Parikh RB et al. *JAMA Network Open.*

eTable 1. Published Electronic Health Record-Based Machine Learning Prognostic Tools

Reference	Population	Outcome	Performance	ML algorithm	Comparator(s)
	**Oncology**				
Elfiky et al, 2018[1]	Patients with cancer beginning chemotherapy at a large academic cancer center	30-day mortality	AUC 0.94	Gradient-boosted trees	None
Bertsimas et al, 2018[2]	Patients with cancer beginning chemotherapy at a large academic cancer center	180-day mortality	AUC 0.83	Decision tree	None
Gupta et al, 2014[3]	Patients with cancer in an Australian regional cancer centre registry	6-, 12-, and 24-month mortality	AUC 0.87 (6-month), 0.80 (12-month), & 0.76 (24-month)	Support-vector machine	Clinician panel
	**General medicine or other**				
Sahni et al, 2018[4]	Minnesota patients admitted to general medicine service	1-year mortality	AUC 0.86	Random forest	Logistic regression
Weng et al, 2017[5]	Cardiovascular disease prediction in UK	10-year cardiovascular event	AUC 0.76	Neural networks, gradient-boosted trees, random forest	Logistic regression, clinical guidelines
Churpek et al, 2016[6]	Hospitalized patients in five hospitals	In-hospital mortality	AUC 0.80	Many	Logistic regression, existing early warning score
Taylor et al, 2016[7]	Adults visiting the emergency department with sepsis	In-hospital mortality	AUC 0.86	Random forest	Logistic regression, classification and regression tree (CART), previously developed prediction tools

© 2019 Parikh RB et al. *JAMA Network Open.*

eTable 2. Variables Included in Machine Learning Algorithms

Laboratories	Units	Features Included
**Age**	Years	
**Gender**	Male/Female	
**Elixhauser comorbidities**	Count	**Count of number times comorbidity was ever coded ("total")**<br>**Count of number of times comorbidity was coded in the past 180 days ("recent")**
Metastatic cancer		
Diabetes, uncomplicated		
Alcohol abuse		
Rheumatoid arthritis/collagen vascular diseases		
Weight loss		
Iron deficiency anemia		
Lymphoma		
Cardiac arrhythmias		
Pulmonary circulation disorder		
Liver disease		
Obesity		
Chronic pulmonary disease		
Hypertension, uncomplicated		
Fluid and electrolyte disorders		
Renal failure		
Other neurological disorders		
Pulmonary valvular disorder		
Peptic ulcer disease excluding bleeding		
Hypothyroidism		
Valvular disease		
Congestive heart failure		
Depression		
Paralysis		
Drug abuse		
Diabetes, complicated		
Coagulopathy		
Blood loss anemia		
Solid tumor without metastasis		
AIDS/HIV		
Hypertension, complicated		
Psychoses		
**EKG Values**		**Count**<br>**First value checked**
T Axis	degrees	

© 2019 Parikh RB et al. *JAMA Network Open.*

R Axis	degrees	**Last value checked**<br>**Maximum Value**<br>**Minimum Value**<br>**Proportion of Values ordered as STAT**
P Axis	degrees	
Q-T Interval	ms	
QRS Duration	ms	
P-R Interval	ms	
QTC Calculation(Bazett)	ms	
Atrial Rate	BPM	
Ventricular Rate	BPM	
**Laboratories**		**Count**<br>**First value checked**<br>**Last value checked**<br>**Maximum Value**<br>**Minimum Value**<br>**Proportion of Values ordered as STAT**
% Basophils Manual	%	
% Eosinophils Manual	%	
Reticulocyte count	%	
% Band Neutrophils	%	
% Lymphocytes Manual	%	
% Eosinophils	%	
% Basophils	%	
% Lymphocyte Variants	%	
% Segmented Neutrophils Manual	%	
% Monocytes	%	
% Lymphocytes	%	
% Myelocytes	%	
% Monocytes Manual	%	
Alkaline Phosphatase	IU/L	
WBC Corrected	THO/uL	
Abs Retic	THO/uL	
# Eosinophils Manual	THO/uL	
# Segmented Neutrophils Manual	THO/uL	
# Basophils Manual	THO/uL	
# Monocytes Manual	THO/uL	
# Atypical Lymphocytes	THO/uL	
# Lymphocytes Manual	THO/uL	
AST	U/L	
Lactate dehydrogenase	U/L	
ALT	U/L	
UA pH	[pH]	
# Band Neutrophils	cells/uL	
MCV	fL	
RDW	fL	
Gamma-Globulin Fract	g/dL	

© 2019 Parikh RB et al. *JAMA Network Open.*

MCHC	g/dL	
Beta-Globulin Fracti	g/dL	
Albumin Fraction	g/dL	
Alpha2-Globulin Fraction	g/dL	
Alpha1-Globulin Fraction	g/dL	
% Nucleated Red Blood Cells	k/cumm	
% Neutrophils	k/uL	
eGFR, AA	mL/min/1.73m2	
eGFR, Non-AA	mL/min/1.73m2	
Magnesium	mg/dL	
Immunoglobulin G	mg/dL	
Immunoglobulin M	mg/dL	
Fibrinogen	mg/dL	
BUN	mg/dL	
Urea Nitrogen	mg/dL	
POC Glucose (Fingerstick)	mg/dL	
Glucose	mg/dL	
Calcium	mg/dL	
Bilirubin, Indirect	mg/dL	
Uric acid	mg/dL	
Bilirubin, Direct	mg/dL	
Phosphate	mg/dL	
Creatinine	mg/dL	
Hemoglobin	mg/dL	
Triglycerides	mm/l	
Total Cholesterol	mm/l	
pCO2 Art	mmHg	
Carbon Dioxide	mmol/L	
Anion Gap	mmol/L	
Lactic acid	mmol/L	
Chloride	mmol/L	
Sodium	mmol/L	
Potassium	mmol/L	
Carcinoembryonic antigen	ng/mL	
Ferritin	ng/mL	
MCH	pg	
INR	ratio	
PTT	second(s)	
PT	seconds	

© 2019 Parikh RB et al. *JAMA Network Open.*

TSH	u[IU]/mL	
Tacrolimus level	ug/L	
# Basophils	x10E3/uL	
# Neutrophils	x10E3/uL	
# Monocytes	x10E3/uL	
# Eosinophils	x10E3/uL	
White blood cells	x10E3/uL	
Platelets	x10E3/uL	
# Lymphocytes	x10E3/uL	
Red blood cells	x10E6/uL	
Albumin	g/dL	
Bilirubin, Total	mg/dL	
Immunoglobulins A	mg/dL	
Protein, Total	g/dL	

Footnote: While all variables in the comorbidity and laboratory sections were included in some form, not all features were included after feature selection. For full list of variables included after feature selection, see https://github.com/pennsignals/eol-onc.

© 2019 Parikh RB et al. *JAMA Network Open.*

eTable 3. Performance Characteristics of Gradient Boosting Model Used for Clinician Surveys

	**AUC**	**Accuracy**	**Alert Rate per 10000 patients**	**F1 Score**	**Negative predictive value**	**Positive predictive value**	**Sensitivity**	**Specificity**
Gradient boosting classifier	0.87	0.952878	0.027268	0.338624	0.967188	0.442396	0.274286	0.984096

© 2019 Parikh RB et al. *JAMA Network Open.*

eTable 4. Distribution of Coded Elixhauser Comorbidities

Comorbidities	Alive at 6 months (N=25460)		Died at 6 months (N=1065)	
	n	%	n	%
Metastatic cancer	4026	15.8	475	44.6
Diabetes uncomplicated	2919	11.5	198	18.6
Alcohol abuse	451	1.8	42	3.9
Rheumatoid arthritis/collagen vascular diseases	1677	6.6	84	7.9
Weight loss	2031	8.0	256	24.0
Deficiency anemia	2232	8.8	85	8.0
Lymphoma	4018	15.8	121	11.4
Cardiac arrhythmias	2473	9.7	214	20.1
Pulmonary circulation disorder	1248	4.9	128	12.0
Liver disease	1487	5.8	114	10.7
Obesity	2771	10.9	97	9.1
Chronic pulmonary disease	3631	14.3	227	21.3
Hypertension, uncomplicated	8600	33.8	472	44.3
Fluid and electrolyte disorders	4526	17.8	417	39.2
Renal failure	1891	7.4	151	14.2
Other neurological disorders	1067	4.2	102	9.6
Pulmonary valvular disorder	1227	4.8	104	9.8
Peptic ulcer disease excluding bleeding	415	1.6	31	2.9
Hypothyroidism	3125	12.3	145	13.6
Valvular disease	1756	6.9	90	8.5
Congestive heart failure	1536	6.0	141	13.2
Depression	3409	13.4	176	16.5
Paralysis	186	0.73	26	2.44
Drug abuse	554	2.2	39	3.7
Diabetes complicated	1032	4.1	79	7.4
Coagulopathy	2692	10.6	212	19.9
Blood loss anemia	572	2.3	27	2.5
Solid tumor without metastasis	13718	53.9	778	73.1
AIDS/HIV	195	0.77	8	0.75
Hypertension, complicated	1395	5.5	120	11.3
Psychoses	288	1.1	19	1.8

© 2019 Parikh RB et al. *JAMA Network Open.*

eTable 5. Baseline Laboratory and Electrocardiogram Values

Value [mean, 95% CI]	Alive at 6 months (N=25460)	Died at 6 months (N=1065)
MCV	90.76 [90.68-90.83]	90.80 [90.34-91.25]
RDW	15.36 [15.33-15.38]	17.06 [16.88-17.25]
MCH	29.89 [29.86-29.92]	29.76 [29.59-29.93]
MCHC	32.94 [32.93-32.95]	32.73 [32.67-32.79]
Hemoglobin	12.16 [12.14-12.18]	11.03 [10.92-11.13]
Hematocrit	36.85 [36.80-36.91]	33.60 [33.28-33.93]
Platelets	227.07 [226.09-228.05]	229.84 [222.39-237.29]
Red Blood Cells	4.09 [4.09-4.10]	3.74 [3.70-3.78]
Creatinine	0.95 [0.93-0.98]	1.03 [0.98-1.08]
White Blood Cells	6.98 [6.88-7.08]	8.03 [7.62-8.43]
Sodium	137.20 [137.18-137.22]	136.24 [136.05-136.43]
Urea Nitrogen	16.83 [16.74-16.91]	18.83 [18.19-19.46]
Chloride	103.79 [103.76-103.82]	102.58 [102.33-102.84]
Potassium	4.13 [4.12-4.13]	4.14 [4.11-4.16]
Anion Gap	7.07 [7.05-7.09]	7.69 [7.54-7.84]
Carbon Dioxide	26.31 [26.29-26.34]	25.94 [25.75-26.13]
Glucose	107.46 [107.10-107.83]	117.00 [114.33-119.66]
Calcium	9.32 [9.32-9.33]	9.15 [9.11-9.20]
ALT	19.97 [19.74-20.21]	26.65 [24.33-28.97]
AST	21.95 [21.74-22.15]	32.16 [29.39-34.93]
Bilirubin, Total	0.55 [0.55-0.56]	0.83 [0.70-0.97]
Alkaline Phosphatase	77.13 [76.59-77.67]	122.28 [114.54-130.02]
# Eosinophils	6.69 [4.87-8.51]	2.83 [1.35-4.31]
# Basophils	1.37 [1.01-1.74]	0.82 [0.08-1.56]
# Monocytes	18.30 [16.02-20.58]	17.77 [5.07-30.48]
# Lymphocytes	101.89 [68.92-134.85]	28.98 [15.31-42.64]
% Monocytes	8.67 [8.64-8.70]	8.79 [8.58-9.00]
% Lymphocytes	22.84 [22.74-22.94]	18.06 [17.46-18.66]
# Neutrophils	142.63 [130.28-154.98]	162.16 [78.66-245.67]
% Eosinophils	2.15 [2.13-2.18]	1.79 [1.66-1.92]
% Basophils	0.57 [0.56-0.58]	0.53 [0.49-0.58]
% Neutrophils	64.88 [64.77-64.98]	70.20 [69.48-70.92]
Albumin	4.03 [4.03-4.04]	3.66 [3.63-3.69]
Protein, Total	6.91 [6.91-6.92]	6.73 [6.68-6.78]
Fingerstick glucose	122.31 [122.11-122.51]	125.22 [123.48-126.96]
MAGNESIUM	1.90 [1.90-1.91]	1.89 [1.87-1.90]
% Nucleated RBC	0.11 [0.07-0.14]	0.50 [0.19-0.80]

© 2019 Parikh RB et al. *JAMA Network Open.*

INR	1.13 [1.13-1.13]	1.18 [1.16-1.20]
PT	13.49 [13.46-13.52]	14.15 [13.90-14.40]
WBC Corrected	6.44 [6.34-6.54]	7.51 [7.03-7.99]
# Band Neutrophils	2.08 [0.54-3.62]	9.54 [-3.68-22.75]
% Basophils Manual	0.08 [0.07-0.08]	0.20 [0.16-0.24]
% Eosinophils Manual	1.12 [1.10-1.13]	1.20 [1.10-1.30]
% Band Neutrophils	0.26 [0.24-0.29]	0.97 [0.80-1.13]
# Atypical Lymphocytes	0.01 [0.00-0.01]	0.01 [-0.00-0.01]
% Lymph Variants	0.03 [0.03-0.04]	0.05 [0.02-0.07]
% Segmented Neutrophils Manual	63.25 [63.14-63.35]	64.79 [63.90-65.68]
% Monocytes Manual	7.15 [7.11-7.18]	7.69 [7.40-7.98]
% Lymphocytes Manual	21.04 [20.94-21.15]	19.66 [18.92-20.39]
# Segmented Neutrophils Manual	3.80 [3.77-3.83]	4.94 [4.62-5.27]
PHOSPHATE	3.40 [3.39-3.40]	3.50 [3.22-3.77]
PTT	28.51 [28.47-28.55]	29.62 [29.26-29.98]
# Basophils Manual	0.01 [0.00-0.01]	0.01 [0.01-0.02]
# Eosinophils Manual	0.05 [0.05-0.05]	0.07 [0.06-0.08]
# Monocytes Manual	0.44 [0.44-0.45]	0.52 [0.48-0.56]
# Lymphocytes Manual	1.49 [1.41-1.57]	1.19 [1.04-1.34]
LACTATE DEHYDROGENASE	199.49 [198.80-200.17]	233.06 [219.03-247.09]
INDIRECT BILI	0.41 [0.41-0.41]	0.49 [0.44-0.54]
Bilirubin, Direct	0.12 [0.11-0.12]	0.27 [0.19-0.35]
URIC ACID	5.04 [5.03-5.04]	5.10 [5.03-5.16]
eGFR, Non-AA	53.30 [53.20-53.41]	51.67 [51.05-52.30]
Ventricular Rate	78.97 [78.88-79.06]	81.75 [80.96-82.54]
R Axis	21.20 [20.99-21.42]	19.53 [17.99-21.08]
QRS Duration	86.89 [86.79-86.99]	87.92 [87.04-88.81]
T Axis	43.32 [43.12-43.52]	44.71 [42.95-46.47]
Q-T Interval	388.86 [388.65-389.07]	383.89 [381.80-385.99]
QTC Calculation(Bazett)	441.30 [441.15-441.46]	442.15 [440.45-443.85]
Atrial Rate	79.56 [79.38-79.74]	84.02 [82.42-85.62]
P-R Interval	153.11 [152.96-153.26]	152.71 [151.74-153.67]
P Axis	51.68 [51.57-51.79]	51.34 [50.55-52.14]
TSH	2.05 [2.02-2.08]	2.19 [2.07-2.30]
UA pH	6.01 [6.01-6.01]	6.05 [6.02-6.08]
UA Specific Gravity	1.17 [1.02-1.33]	1.02 [1.02-1.02]
eGFR, AA	55.86 [55.74-55.98]	54.44 [53.75-55.13]
LACTIC ACID	1.11 [1.10-1.11]	1.15 [1.13-1.17]
IMMUNOGLOBULIN G	862.30 [859.53-865.06]	876.27 [854.87-897.67]

© 2019 Parikh RB et al. *JAMA Network Open.*

% Myelocytes	1.07 [1.06-1.08]	1.28 [1.20-1.36]
IMMUNOGLOBULIN M	51.22 [49.79-52.64]	46.45 [45.20-47.70]
FERRITIN	200.41 [196.32-204.49]	369.52 [305.59-433.46]
IMMUNOGLOBULINS A	96.27 [95.09-97.44]	117.54 [97.39-137.69]
RBC	4.00 [4.00-4.00]	3.92 [3.90-3.95]
WBC	6.41 [6.38-6.44]	6.69 [6.50-6.88]
Triglycerides	114.15 [113.75-114.55]	116.17 [114.00-118.35]
CARCINOEMBRYONIC ANTIGEN	6.26 [5.52-7.01]	57.65 [16.77-98.53]
BUN	16.20 [16.16-16.24]	17.37 [16.87-17.86]
Albumin Fraction	3.90 [3.90-3.90]	3.87 [3.86-3.88]
FIBRINOGEN	371.21 [370.94-371.48]	373.57 [370.19-376.96]
RETICULOCYTE COUNT	2.23 [2.22-2.24]	2.25 [2.21-2.29]
Abs Retic	74.50 [74.31-74.69]	73.95 [73.17-74.72]
Total Cholesterol	171.57 [171.39-171.76]	170.57 [169.48-171.65]
Alpha2-Globulin Frac	0.80 [0.80-0.80]	0.80 [0.80-0.80]
Beta-Globulin Fracti	0.71 [0.71-0.71]	0.72 [0.70-0.73]
Gamma-Globulin Fract	0.91 [0.91-0.91]	0.91 [0.90-0.92]
Alpha1-Globulin Frac	0.30 [0.30-0.30]	0.31 [0.30-0.31]
pCO2 Art	39.01 [39.00-39.02]	38.97 [38.86-39.07]
TACROLIMUS (FK506)	5.70 [5.70-5.71]	5.72 [5.68-5.76]

Footnote: All laboratory values are the last value documented prior to the unique encounter for each patient

© 2019 Parikh RB et al. *JAMA Network Open.*

eTable 6. Variable Importance by Model, Top 20 Predictors

**Logistic Regression**
Albumin: last
Solid tumor: recent count
Metastatic cancer: recent count
Patient age
Alkaline phosphatase: last
Gender
Solid tumor: total count
Blood loss anemia: total count
Red blood cells: last
MCHC: last
BUN: last
Fluid and electrolyte disorders: recent count
Other neurological disorders: recent count
Blood loss anemia: recent count
Lymphoma: recent count
Calcium: last
Drug abuse: total count
Q-T interval: last
Pulmonary circulation disorders: recent
Weight loss: recent
**Random Forest**
Metastatic cancer: recent count
Albumin: last
Alkaline phosphatase: last
Albumin: minimum
Patient age
Alkaline phosphatase: maximum
Solid tumor: total count
Solid tumor: recent count
Metastatic cancer: total count
% lymphocytes: minimum
% lymphocytes: last

© 2019 Parikh RB et al. *JAMA Network Open.*

RDW: maximum
% neutrophils: last
Alkaline phosphatase: standard deviation
Magnesium: count
Magnesium: standard deviation
RDW: last
MCV: count
AST: last
RDW: standard deviation
**Gradient Boosting**
Albumin: last
Solid tumor: recent count
Metastatic cancer: total count
Metastatic cancer: recent count
Alkaline phosphatase: last
% lymphocytes: last
% neutrophils: last
Albumin: minimum
Alkaline phosphatase: maximum
% lymphocytes: minimum
RDW: last
Patient age
MCV: standard deviation
% neutrophils: maximum
Solid tumor: total count
Albumin: maximum
% lymphocytes manual: minimum
Sodium: minimum
ALT: count
Alkaline phosphatase: standard deviation

© 2019 Parikh RB et al. *JAMA Network Open.*
